# Guidelines and quality criteria for artificial intelligence-based prediction models in healthcare: a scoping review

**DOI:** 10.1038/s41746-021-00549-7

**Published:** 2022-01-10

**Authors:** Anne A. H. de Hond, Artuur M. Leeuwenberg, Lotty Hooft, Ilse M. J. Kant, Steven W. J. Nijman, Hendrikus J. A. van Os, Jiska J. Aardoom, Thomas P. A. Debray, Ewoud Schuit, Maarten van Smeden, Johannes B. Reitsma, Ewout W. Steyerberg, Niels H. Chavannes, Karel G. M. Moons

**Affiliations:** 1 Department of Information Technology and Digital Innovation, Leiden University Medical Center, Leiden, The Netherlands; 2 Clinical AI Implementation and Research Lab, Leiden University Medical Center, Leiden, The Netherlands; 3 Department of Biomedical Data Sciences, Leiden University Medical Center, Leiden, The Netherlands; 4 Julius Center for Health Sciences and Primary Care, University Medical Center Utrecht, Utrecht University, Utrecht, The Netherlands; 5 Cochrane Netherlands, University Medical Center Utrecht, Utrecht University, Utrecht, The Netherlands; 6 National eHealth Living Lab, Leiden, The Netherlands; 7 Department of Public Health and Primary Care, Leiden University Medical Center, Leiden, The Netherlands

**Keywords:** Medical research, Epidemiology

## Abstract

While the opportunities of ML and AI in healthcare are promising, the growth of complex data-driven prediction models requires careful quality and applicability assessment before they are applied and disseminated in daily practice. This scoping review aimed to identify actionable guidance for those closely involved in AI-based prediction model (AIPM) development, evaluation and implementation including software engineers, data scientists, and healthcare professionals and to identify potential gaps in this guidance. We performed a scoping review of the relevant literature providing guidance or quality criteria regarding the development, evaluation, and implementation of AIPMs using a comprehensive multi-stage screening strategy. PubMed, Web of Science, and the ACM Digital Library were searched, and AI experts were consulted. Topics were extracted from the identified literature and summarized across the six phases at the core of this review: (1) data preparation, (2) AIPM development, (3) AIPM validation, (4) software development, (5) AIPM impact assessment, and (6) AIPM implementation into daily healthcare practice. From 2683 unique hits, 72 relevant guidance documents were identified. Substantial guidance was found for data preparation, AIPM development and AIPM validation (phases 1–3), while later phases clearly have received less attention (software development, impact assessment and implementation) in the scientific literature. The six phases of the AIPM development, evaluation and implementation cycle provide a framework for responsible introduction of AI-based prediction models in healthcare. Additional domain and technology specific research may be necessary and more practical experience with implementing AIPMs is needed to support further guidance.

## Introduction

Prediction models have a prominent role in healthcare research and practice. Diagnostic prediction models make predictions about the current health status of a patient, whereas prognostic prediction models estimate the probability of a health outcome in the future^1,2^. Methods from the machine-learning (ML) domain and its broader field of Artificial Intelligence (AI) have seen a rapid increase in popularity for prediction modeling. While the opportunities of ML and AI in healthcare are promising, the growth of complex data-driven prediction models requires careful quality and applicability assessment to guarantee their performance, safety and usability before they are used and disseminated in practice.

A framework for structured quality assessment across the entire AI-based prediction model (AIPM) development, evaluation and implementation cycle is still missing. Such a framework is needed to ensure safe and responsible application of AIPMs in healthcare. For example, it can provide guidance on the appropriate validation steps needed before implementation to prevent faulty decision making based on overfitted models. The absence of such a framework may have contributed to relatively few models having been implemented to date^3^. We define the term AI-based prediction model (AIPM) as follows: a data-driven model that provides probabilistic patient-level predictions of the current presence or future occurrence of a certain outcome (e.g., a certain patient condition), given certain input (e.g., certain patient characteristics, genetic markers, medical images, or other types of features).

We aimed to identify existing guidelines and quality criteria regarding six predefined phases of the AI-based prediction model development, evaluation and implementation cycle. The six AIPM development phases range from preparation and data collection to implementation in daily healthcare practice (see Box 1) and form the core structure and driver for this review. These phases are based on the predominant phases in clinical prediction model research^4,5^. We performed a scoping review to outline the most important aspects to consider in each phase, while providing pointers to relevant guidelines and quality criteria in the recent literature, focusing on actionable guidance for those closely involved in the AIPM development, evaluation and implementation cycle (e.g., software engineers, data scientists, but also health professionals). We also aimed to identify gaps in the existing guidance.

Box 1 Phases^1^ of AI prediction model construction *Phase 1. Preparation, collection, and checking of the data:* the preparation, collection and checking of the data to facilitate proper AIPM development (phase 2) and AIPM validation (phase 3).

*Phase 2. Development of the AIPM:* the modeling of the relation between the predictive input variables (features/predictors) and the health outcome of interest, via a mathematical formula or algorithm.

*Phase 3. Validation of the AIPM:* the testing (validating) how well the developed AIPM from phase 2 predicts the outcome in individuals whose data were not used during AIPM development (so called external validation data), quantifying the AIPM’s predictive performance.

*Phase 4. Development of the software application:* the development of the software application, containing the programming, design, usage and support of the digital packaging of the AIPM.

*Phase 5. Impact assessment of the AIPM with software:* the assessment of the impact of the usage of the AIPM and software on daily healthcare practice, patient or individual health outcomes, and healthcare costs.

*Phase 6. Implementation and use in daily healthcare practice:* the implementation of the AIPM in routine care, including maintenance, post-deployment monitoring, and updating.

^1^These phases are primarily introduced to provide a clear structure to the article. In practice, the order of these phases may slightly differ.

## Methods

**Fig. 1:**
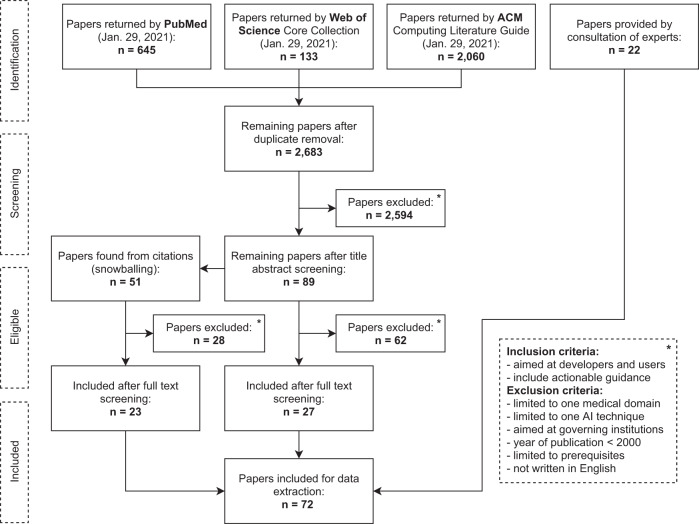
Flow diagram of screening strategy. This flow diagram displays the screening strategy for the inclusion of guidance documents in this scoping review.

A multi-stage screening strategy was used for this scoping review driven by the six AIPM development phases (Fig. 1). We searched for relevant academic literature published from January 2000 up to January 2021 in three online databases containing a variety of medical, technical, ethical, and social science literature: PubMed, Web of Science, and ACM Digital Library. The search strings consisted of a combination of search terms related to: (i) guidelines, quality criteria, best practices and reporting standards (ii) artificial intelligence, including machine-learning and prediction modeling in general and (iii) topics relating to one of the six phases of AIPM development (see Box 1), such as ‘data cleaning’ for phase 1 and ‘impact assessment’ for phase 5. For the complete search strings, and a filled PRISMA reporting checklist for scoping reviews, see Supplementary Tables 1 and 2, respectively.

We used the following inclusion criteria for our review process: (i) documents (e.g., reports, articles, or guidelines) primarily aimed at the individuals directly involved with the development, evaluation, and implementation of AIPMs (excluding institution or organization wide guidance) and (ii) documents with actionable guidance (e.g., clearly defined recommendations on how to develop AIPMs and implement them into practice). The following exclusion criteria were used: (i) guidance limited to one medical domain (e.g., cardiology) without generalizing to other domains, (ii) guidance limited to one AI technique (e.g., reinforcement learning) without generalizing to other techniques, (iii) guidance aimed at governing institutions, (iv) documents published before 2000, (v) guidance limited to the prerequisites to develop, validate and implement an AIPM (e.g., documents focusing on the development of data infrastructures or legal and governance frameworks), and vi) documents not written in English.

Two reviewers (AdH and AL) performed title and abstract screening of the documents produced by the online database search. Additional literature was added through manually scrutinizing (snowballing) the reference lists of the identified documents. We also asked a convenience sample of 14 AI experts from academia and industry to provide potentially relevant sources (see Supplementary Table 3). These additional search strategies were specifically aimed at identifying gray literature consisting of government, institutional or industry documents and websites. The two reviewers performed a full-text screening on all retained literature (including gray literature). Conflicts regarding the eligibility of documents during the screening process were resolved by consensus in regular sessions between the two reviewers.

For the data extraction, two reviewers (AdH and AL) independently identified keywords from each included document which represented the area on which guidance was provided (e.g., development, parameter tuning). Each keyword was mapped to more central topics pertaining directly to the AIPM development phases (e.g., development and parameter tuning were mapped to AIPM training). When applicable to more than one phase, the keyword was placed in a phase-overarching topic (e.g., algorithmic bias). The mapping was adjusted and fine-tuned repeatedly over the course of data extraction and validated based on the input from three co-authors (IK, SN, and MvS). During a second full-text screening round, all identified guidance was extracted according to the topics, summarized, and placed in the review section corresponding to that phase-specific or phase-overarching topic.

## Results

After removing duplicates, the search resulted in 2683 documents. The title and abstract screening reduced this number to 89 documents. Snowballing added 51 documents. A total of 27 papers from online databases, 23 from manual inclusion and 22 from expert consultation, were retained after full-text screening. This led to a total of 72 documents included in the review (Fig. 1). Data extraction resulted in 138 keywords, which were mapped to 27 phase-specific topics and 6 phase-overarching topics (see Supplementary Table 4). In the next sections, the summarized guidance is structured per phase. The phase-overarching topics are summarized in Box 2 and further integrated in the phase-specific summaries (as shown in Supplementary Table 5). Supplementary Table 6 can be used as a lookup table structuring the hyperlinks to the identified guidance per phase and supplementary Table 7 provides the affiliations (industry, academia, governing), geographical region and type of source (literature search, snowballing, expert consultation).

Box 2 Descriptions of identified phase-overarching topics^2^ *Algorithmic bias* refers to an AIPM that systematically disadvantages individuals belonging to a particular subgroup when there is no a priori medical justification for this discrepancy^22–25,61–63,72,91^. Subgroups can for example be based on gender, race, culture, religion, age, sexual orientation, socioeconomic background, ability status and ethnicity^6,7,22–24,26,32,42,61,72,79,91^. There are two important causes for algorithmic bias: non-representative development data^8,16,19,20,22–27,43,46,56,58,59^ and historical human biases that are reflected in data^22–25,62^. The field of AI fairness aims to address algorithmic bias by studying how best to identify and mitigate it^23,43^.

*Transparency and openness* entail the possibility to inspect sufficient details on e.g., study design, data selection, analytical scripts, the AIPM model and modeling approach, justifications, and limitations, in a way that could allow others to reproduce the process (e.g., for independent external validation of the AIPM)^9,22,40,58,62,125,126^. Recommendations regarding transparency often involve detailed reporting, following relevant reporting guidelines^6,8,37,50,64,65,72^, and sharing of relevant information, code, and data across the different phases.

*Interpretability* of an AIPM refers to the degree to which a human can understand how an AIPM comes to its predictions or classifications^75^. Being able to interpret an AIPM may facilitate detection of potential errors and biases in its predictions^7,8,27,61^. This may be an important factor in obtaining trust and acceptance by end users (e.g., healthcare professionals and patients)^10,24,26,40,47,72,73,87^. Interpretability and transparency are closely related. For example, an interpretable AIPM may allow a physician to be more transparent about the decision-making process to patients^16,22,40,41,62,87^.

*Team members, end users, and stakeholders* should be considered carefully throughout the AIMP lifecycle (see Box 1). It has been recommended that already from the start the AIPM development team must cover a multidisciplinary technical, methodological and medical expertise^8,11,17,20,23,26,32,71^, consider data and project management^8,11,18,20,26^, and attend to the diversity of the anticipated end users of the AIPM^11,20,26,62,66^. Identifying and involving the right expertise and stakeholders in each consecutive phase of the AIPM development, evaluation and implementation cycle is crucial for its success in daily healthcare practice^8,18,22,23,26,32,58,79,88^.

*Security* encompasses the protection of the AIPM and its (personal) data against malicious actors^9,22,32^. Two risks particularly concerning an AIPM are the misuse of the (often large amounts of) development and validation data^23,33^ and software vulnerabilities introduced by the new AIPM code and infrastructure^23^. Security measures protecting against these vulnerabilities form part of the AIPM architecture and should be tested before deployment^32^.

*Risks* refer to any (unintended) consequences of the AIPM’s application that threaten the AIPM’s safe and effective application^9,18,20,57^. Potential risks are flaws in the design of the AIPM, technical defects, inappropriate or malicious use, process changes, security breaches (see Security above), and disparate outcomes for different use cases or subgroups (see algorithmic bias and fairness above)^12,26,28^. Safety (for patients and healthcare professionals) should be considered during all phases of AIPM development^20^.

^2^An index on where each phase-overarching topic is further discussed in the article can be found in Supplementary Table 5.

### Phase 1. Preparation, collection, and checking of the data

#### Medical problem and context

One of the very first aspects of developing and validating an AIPM as recommended in literature is to clearly specify the medical problem and context that the AIPM will address, and to identify the healthcare setting(s) in which the AIPM is to be deployed^3,6–15^. Before starting actual AIPM development, it is advocated to first conduct a thorough investigation into the current standard of care, context and workflow^7–11,14–18^, and to provide a clear rationale for why the current approach falls short. For example, via analysis of the needs of targeted end users through observations and interviews, and by involving them from the start in the developmental process^11,12,17–20^. Once a precise (diagnostic or prognostic) prediction task has been formulated, healthcare actions, treatments or interventions should be defined that are to follow from the AIPM predictions^3,6–8,10,11,13,17,21^. Clinical success criteria must be determined and described^3,6,7,9,11,12,20,22^, including an analysis of the potential risks of prediction errors^6,23^. Developers are advised to perform a feasibility check to assess at an early stage whether the expected benefit of the AIPM to the healthcare system outweighs the costs of developing the AIPM, its maintenance, and other consequences of incorrect (or unfair) use of the predictions of the AIPM^9–12,22,24–28^.

#### Patient privacy

The literature advocates that, before starting data collection, the development team should ensure compliance with relevant privacy legislation (e.g., General Data Protection Regulation (GDPR)^29^, the Personal Information Protection and Electronic Documents Act (PIPEDA)^30^ or the Health Insurance Portability and Accountability Act (HIPAA)^31^) and take measures to protect the privacy of the individuals whose data are used for AIPM development, evaluation, or application^8,12,20,23,26,32–36^. Consultation with data protection specialists has been recommended^23^. Legislation may require identification of the right legal basis (such as informed consent) for processing confidential information of individuals^12,20,26,32,33,36,37^. In many cases, individuals must be informed about the processing of their personal data^20,23,29,35,36,38^. In the case of using (existing) data that was originally collected for a purpose unrelated to the AIPM (e.g., patient care), there must be an adequate processing basis for re-using these data for AIPM-related purposes^23,35^. The legal basis can be different for the development and validating versus deployment phases of AIPMs^23,33^. More specifically, data subjects may not be directly affected by AIPM development but are often affected by AIPM deployment as the AIPM’s predictions could influence the treatment decisions of data subjects. Depending on local legislation, it can be required (e.g., under GDPR^29^ or the Canadian Privacy Act^39^) to develop a data protection impact assessment^23,26,32,33,35,40,41^, assign a data protection officer^23,26,36^, and take measures to conduct data protection oversight, by limiting access only to necessary and qualified personnel^23,26,35^. Moreover, taking measures to achieve privacy by design^12,23,26,32,35,36,41–43^, such as data minimization^23,35,41^, encryption^35,41^, or the use of data pseudonymization or anonymization methods is recommended^35,41^. The use (or absence) of such methods should be clearly motivated^8,12,14,20,26,35,44^, especially whenever patient data leave primary care systems^8^. Any trade-offs between predictive performance and privacy should be considered^23^. Finally, under some data protection regulations, individuals have the right to withdraw consent, the right to object, and the right to be forgotten (e.g., under GDPR^29^ and the California Consumer Privacy Act^45^), which should be considered and implemented throughout development and deployment stages of the AIPM^12,23,36,41^.

#### Sample size

It is recommended that the amount of collected data is sufficiently large for the intended purpose^6,8,12,15,20,22,26,46–49^, is ideally prespecified^8^ and should be clearly reported^3,14,37,46,50^. The required sample size for AIPM development depends on the specific context, including the used prediction modeling method, the number of features, the proportion of the predicted health outcome (in case of categorical outcomes), and the desired predictive performance^47,48^, which may be linked to a minimal required clinical impact^8^. For regression-based methods^48^, and a selection of machine-learning-based methods^47^, technique-specific a priori sample size calculations are available, although for many model architectures and settings (e.g., semi-supervised learning, decision trees, or convolutional neural networks) no specific guidance was found. If some (closely related) data are already available, it has been suggested to inspect the model’s learning curve in that data, setting out prediction performance against the amount of used data, to estimate the required total sample size for a specific use case^47,51,52^. For external predictive performance evaluation (discussed in more detail in phase 3), as a rule of thumb, it has been suggested that the sample should at least contain 100 events per outcome^53^, but for binary and continuous outcomes more specific sample size calculations are now available^54,55^.

#### Representativeness

The literature recommends that the collected data are representative of the target population and intended healthcare setting, and sufficiently cover the relevant real-world heterogeneity and diversity^7,9,12,26,27,32,37,48,56,57^. This representativeness criterion is considered crucial to assess and combat algorithmic bias^8,16,19,20,22–27,43,46,56,58,59^ and poor calibration^60^. Thorough assessment of the representativeness of the data is strongly advised^6,7,13,14,16,26,37,46,56,57^, for which a detailed description of the collected data is required, including the time span of data collection^3,6,7,9,12,21,22,37,61^, the collection site and setting^3,7,14,15,20–22,24,42,46,61–63^, relevant population characteristics such as gender, age, ethnicity, and relevant medical history^3,7,15,21,37,46^, and any inclusion or exclusion criteria that were used^3,6,7,9,13–16,20,21,37,50,56,64,65^. Finally, revaluation and reporting of any differences between the collected data and the intended target population and setting is emphasized^3,6,13,14,16,24,26,46,56,57^, including which groups may be underrepresented in the data with respect to the target population.

#### Data quality

Extensive assessment of data quality has been widely recommended^6,7,12,13,16,22,24,26,33,37,64,65^. For both feature variables as well as outcomes, this involves the inspection and description of missing data, consideration of potential errors in measurement, and their underlying mechanisms (e.g., random or systematic)^3,6,9,13,15–17,20,22,27,37,46,47,66,67^. A clear definition of how and when each variable was measured should be provided^3,6,9,12–15,17,21,22,25,37,46,50,58,62,64,65^, including specification of measurement instruments or tools (e.g., make and model of devices). Any known data quality risks and limitations should be reported and related to potential impact on the AIPM’s predictions and its validation (with special attention to algorithmic bias)^3,13,20,22,26,32,33,37,43,57^. An additional validity check could be performed by randomly sampling a portion of the data and manually checking it for errors^28,61^. The proportion of errors should be reported^61^. The literature also recommends the installation of a process through which data errors can be corrected^43,61^. Note that when such a process is installed, it should also be employed during implementation and not just during model development. It must be clearly identified whether data were collected retrospectively or prospectively^6,14,15,21,46^. Prospective data collection may be preferred as it more closely matches the real-world operating conditions^56^. It was pointed out that one should be aware of potential quality risks of routinely collected data as such data are often collected for a different purpose^56,68^.

The literature places a particular emphasis on the quality of outcome data, more specifically the reference standard or ‘ground truth’. A clear rationale on outcome data collection needs to be provided (e.g., an expert panel, biopsy, clinical determination via laboratory tests), and any potential quality issues^3,6,13–15,21,46^. In case the outcome data were manually labeled, the AIPM development and validation team are urged to precisely specify how and by whom data were labeled, including the level of experience of the labelers, and elaborate on relevant pitfalls or difficult cases^8,10,15,21,46,64,65,67^. Ideally, to ensure label quality and prevent bias in AIPM evaluation, it was advised that this is a well-defined and controlled process^46,66^, where experts labeling the data work independently from each other^8,21^, and are *not* directly involved in performance assessment of the AIPM^15,46^. Depending on the exact procedure, inter-observer variability or test reproducibility^8,15,21,46^ should be calculated to obtain an assessment of label quality.

#### Data preprocessing

To prepare data for the consecutive phases, or handle identified data quality issues, data preprocessing steps may be applied. Such preprocessing steps can include splitting the data into different subsets (e.g., train, tuning, and test sets), augmenting data, removing outliers, re-coding or transforming variables, standardization, and imputation of missing data^6,13,17,27,46,47,49,68^. The literature stresses that detailed description of any preprocessing steps applied to the raw data should be provided, including software used to perform the processing steps^3,6,7,9,13–15,22,50,61,62,64,65^. Missing data imputation is generally recommended over complete case analysis where incomplete data are excluded, but this should depend on the underlying missing data mechanism (missing completely at random, missing at random, or missing not at random)^13,17,47,49,68^. Any data augmentation should be carefully considered against the potential introduction of bias, and model developers are advised to collaborate with domain experts on these preprocessing steps^9,22,46^. Finally, the literature stresses that data splitting actions, must happen *before* any other preprocessing steps are applied (e.g., missing data imputation or standardization)^27,69,70^. This is crucial to prevent information leakage between data subsets, which leads to overoptimistic AIPM predictive performance.

#### Data coding standards

To facilitate interoperability, and easier adoption of the AIPM into healthcare settings, it has been recommended to align data management with relevant coding standards and widely adopted protocols^20,26^. Relevant standards may include SNOMED CT for coding clinical data, ICD-10 and OPCS4 for clinical conditions and procedures^20^. Additionally, adopting data exchange protocols in the final AIPM software design has been recommended, but is discussed later in the article (in phase 4, about development of the software application).

### Phase 2. Development of the AIPM

#### Model selection and interpretability

The literature indicates that the following aspects may affect the choice for a certain modeling technique (e.g., regression, decision tree, neural network): prediction performance, interpretability, the familiarity of the modeling technique to the end user, computational requirements, development and validation costs, maintenance, privacy, sample size, and the structure of the data^6,9,13,16,17,22,23,71^. It is recommended that any motivations for choosing a modeling technique should be clearly articulated^6,8,13,14,20,23,24,26^, including benefits and potential risks associated with the chosen technique^13,16,20,23,24,26,32^. Facilitating interpretability of the AIPM, e.g., by providing insight into the impact of each feature or predictor on the predicted outcome^6,14,16,47,57,72,73^, is frequently mentioned as an important aspect for AIPM acceptance into healthcare practice^10,24,26,40,47,72^. Important to note is that the term AIPM interpretability—in this scoping review - does not imply causal interpretability (e.g., high feature impact does not imply causal influence of that feature on the actual health outcome). Interpretability may help to detect trivial and erroneous AIPMs^7,27^, provide medical domain experts with a possibility to discuss whether the associations on which the AIPM relies are likely to remain stable^8,27,61^, help to identify algorithmic bias^7,22,24,27,40,43^, provide information on where the AIPM could be most easily attacked^27^, or how the AIPM may behave under dataset shift^7^. Neural networks are for example recommended for high volume, dense, and complex data types^13,74^, but they are also considered black boxes^23,24,33^, for which additional model-agnostic interpretation tools (explainable AI) are needed to give insight into the importance of individual features for the predictions^13,23,24,33,57,75^. This is in contrast with linear regression and decision trees, which have been considered inherently interpretable approaches. Irrespective of the modeling choice, facilitating interpretability is generally encouraged^13,23,24,32,33,40,41,57,62,71^, in particular when AIPMs rely on sensitive social and demographic data, or if the AIPM’s predictions significantly affect healthcare decision making and a patient’s treatment^16,22,41^. Moreover, under the GDPR^29^, patients have a right to an explanation that enables them to understand why a particular decision was reached^36,40,41^. If a form of interpretability is required, the underlying reasons should be made explicit^9,40^.

#### Training the AIPM

Training (or fitting) the AIPM is the process of determining the values of any model parameters (e.g., also called weights, or coefficients) of the AIPM. Beside model parameters, AIPM development involves choosing hyperparameters, which influence model training and design, but are not necessarily part of the AIPM itself (e.g., penalization factors of shrinkage, learning rates, or the depth of tree-based methods). Automatic optimization of hyperparameters (also referred to as *tuning*) has been recommended^9,27,66,76,77^, for example, via nested cross-validation, or using a small representative held-out tuning dataset. To foster transparency and replicability it is advised that any details about training and hyper-parameter optimization procedures should be reported, including the final values of the (hyper-)parameters, the number of intermediate models trained to come to the final model, and an evaluation of predictive performance on the training data^3,8,13–15,50,61^.

#### Internal validation

The goal of internal validation is to assess the predictive performance of an AIPM in data that are unseen with respect to model training but come from the same population and setting.

To assess AIPM performance, the literature stresses that data should be strictly separated into training, tuning and test sets^7,8,13,77^, possibly stratified by the outcome event^9,27^ to prevent data leakage, which can result in optimistically biased evaluation^7,13,27,69^. Here, the training data is used to train the AIPM, the tuning data for optimizing the hyperparameters, and the test data for assessing the AIPM model performance. Variations on the simplistic ‘split sample’ validation have been suggested for better data efficiency and heterogeneity assessment (e.g., k-fold cross-validation or bootstrapping). Especially for small datasets, a cross-validated procedure is recommended^13,27^. The cross-validated procedure should incorporate all processing steps (standardization, imputation etc.) on the data to prevent data leakage^9,69^. The split of the data and any potential repeats of this splitting procedure should be reported^13,14,50^.

Following the literature, the performance evaluation should be based on at least discrimination and calibration^5,6,9,13,17,49,56,78^. Discrimination refers to the ability of the AIPM to distinguish between subjects with and without the outcome of interest. It is recommended to define the metrics used to measure discrimination prior to the validation^6,8,13^. The chosen metrics should correspond with the intended medical use and should be chosen in close collaboration with domain experts (e.g., an AIPM estimating the risk of breast cancer should be highly sensitive)^7–9,14–16,19,57,79,80^. Discrimination is commonly quantified by the area under the receiver operating characteristic curve^9,15,17,46,49,56,69^. In the case of a clearly defined probability threshold, other metrics could also be used like sensitivity (also labeled: ‘recall’) and specificity, or the positive and negative predictive value (also precision)^9,10,19,72,80^. Note that fixed probability thresholds are not always considered necessary and when they are, they should be carefully determined in collaboration with medical experts^81^.

Calibration refers to the concordance between predicted and observed probabilities. A calibration plot is the recommended method to evaluate calibration^6,17,49,56,60^. Discrimination and calibration evaluation metrics should be documented for all datasets^13,14,16^. It is recommended to calculate confidence intervals to accompany these metrics^8,10,14,15,21,22,24,27,46,61^.

For some application types, Decision Curve Analysis (DCA) is considered a valuable addition to the discrimination and calibration of the AIPM. This performance assessment quantifies how the AIPM could impact patient care within the relatable workflow. Unlike discrimination and calibration, DCA derives the clinical utility from the predictive performance^5,6,17,49,68,72^. Promising results in a DCA can provide a clear indication that an AIPM could benefit daily healthcare practice. It could therefore serve as a precursor (but not a replacement) of a prospective impact study or more fully developed cost-effectiveness analysis (see phase 5).

#### Measures to reduce risk of overfitting

If an AIPM is adapted too much to the training data, and therefore its predictions no longer generalize well to new individuals not used for the development of the AIPM, the model is said to be overfitted^8,47,56,60,76,78^. Often mentioned factors contributing to overfitting are a small sample size in combination with many candidate features, perfect separation on rare categories, and a large imbalance resulting in a small number of events for one of the outcomes^6,47,49,72,76,77,82^. To prevent overfitting, a multitude of strategies are available, often aimed at reducing AIPM complexity. It has been widely recommended to report any measures taken to prevent overfitting^3,7,8,13,15^. One commonly referred strategy is feature selection^13,15,27,47,76^, for which it is explicitly recommended that selection should work independently of model training (unlike in methods as forward and backward selection) and is best informed - a priori - by medical expert knowledge or existing literature^13,17,76^. Other suggested strategies to combat overfitting are dimensionality reduction^47,76^, which can be implicit (e.g., common in neural networks)^76^, and explicit penalization of complexity (e.g., regularization)^17,49,76^. It should be noted that when the sample size is simply too small, even penalization methods have been shown ineffective to mitigate overfitting^83,84^.

#### Measures to identify and prevent algorithmic bias

The literature indicates that tools to identify and mitigate algorithmic bias should also be developed in the AIPM development phase when applicable. First, a definition of fairness should be chosen that corresponds with the AIPM’s intended use^16^. This definition should be integrated with model development as part of the AIPM’s evaluation metrics^22,24,25^. Examples of fairness metrics are outcome parity^22,23,25,42,43^, true (false) positive (negative) rate parity^22,23,25,42,43,79^, positive (negative) predictive value parity^22,42,43^, individual fairness^22^, counterfactual fairness^22,24,42,59^, and equal calibration^23^. Developers are advised to make the chosen fairness metrics available in a Fairness Position or Bias Impact Statement that is reviewed by stakeholders^22,23,25,26,62^. They are also advised to avoid modeling techniques for which it is altogether impossible to evaluate algorithmic bias in an AIPM, for example due to the high dimensionality of its architecture^22^.

Upon identification, algorithmic bias should be addressed by employing an appropriate mitigation strategy during AIPM development, which may be different for different applications and domains. When the bias is caused by unrepresentative training data, the main recommendation is to redo the data collection to rectify this^8,16,19,20,22–27,43,46,56,58,59^. Unrepresentative training data may also be addressed by undersampling the overrepresented group or oversampling the underrepresented group^23,42^. However, this may cause miscalibration of the model predictions and should be used with caution^85^. The most popular recommendation addressing other causes of algorithmic bias (e.g., historical human biases reflected in the data) is to exclude or reweigh the features causing the algorithmic bias^22,23,25,27,43^, although this may not eliminate the bias altogether. Alternatively, the predictions themselves can be reweighed by adjusting the probability threshold per subgroup^42,43^. Lesser mentioned recommendations consist of the application of fairness optimization constraints during AIPM training^42,43^ and the development of separate models per specific subgroup^23^.

Note that the preconceptions and biases of designers can be replicated in their modeling choices^22^. It is therefore considered important to compose a diverse development team^17,22–25^, create awareness and involve stakeholders in design choices^22,24,26,27,72^. Also, developers should keep evaluating algorithmic bias at every stage of the development process^32^.

#### Transparency of the modeling process

The literature advocates that the final AIPM structure should be described in detail, covering input, outputs, and all intermediate layers or parameters^3,14,15,50^. To facilitate transparency and reproducibility of the developmental process, the used computational architecture, high-performance techniques, software packages, and versioning (data, model, configurations and training scripts) should be reported^13,14,16,50,64–66^. Code for the complete model building pipeline should be published in well-documented scripts with computer environment requirements when possible^7,8,13,14,16,19,20,24,25,27,33,50,62,64,65^, including statements about any restrictions to access or re-use.

### Phase 3. Validation of the AIPM

#### External performance evaluation

In practice, an AIPM is likely to be applied in a setting that differs from the setting in which the AIPM was developed, which may have an impact on AIPM performance. In contrast to internal validation (phase 2), external validation is the application of an existing model without any modifications to data from a different population or setting compared to model development (see Generalizability below). The literature highly recommends external validation for all AIPM applications when applied to a new setting^3,9,17,49,86^. Similar to internal validation of the AIPM, external AIPM model validation can be based on discrimination (area under the receiver operating characteristic curve, sensitivity, specificity, positive and negative predictive values), calibration (calibration plot)^5,6,13,17,49,56,78^, and Decision Curve Analysis^5,6,17,49,68,72^. When possible, the literature recommends the comparison of current best practice (e.g., an existing prediction model or medical decision rule) to the AIPM performance^7,8,14–16^.

External validation can be performed on retrospective or prospective data. Although prospective validation is rare, it is preferred by the literature^5,14,56^, as it provides a better idea of the AIPM’s true applicability to medical practice and allows the healthcare professionals to identify and review errors in real time^19,72^. External validation is ideally performed by independent researchers from other institutions or settings^3,8,10,16,27,68,72^. The necessity for external validation by independent researchers may depend on the risks posed by the application (for example based on the level of autonomy of an AIPM)^80^.

#### Generalizability

Generalizability refers to the AIPM’s ability to generalize its performance to a new setting. Poor generalizability may be caused by overfitting (see phase 2) or development data that were unrepresentative for the new setting (see phase 1). The literature recommends to assess generalizability on external data from a different time period, place, or healthcare setting^3,7,8,10,16,17,27,56,68,72,79^.

To ensure the generalizability of the AIPM to the intended healthcare setting, developers are advised to extensively validate the model for representative data from that setting^6–8,10,13–15,24,27,56,64,66,68,72,77,79,87,88^ (see phase 1, Representativeness). The intended healthcare setting may be different from the population or setting on which the AIPM was originally developed (e.g., an AIPM developed at a tertiary care center applied to a smaller hospital). It is advised that the size of this validation data should follow the available sample size recommendations for AIPM validation (see phase 1)^53–55^. Developers are urged to clearly describe any differences between the development and validation data where possible^14^ and report other sources potentially affecting generalizability^6,8,27^. Still, AIPM updating, site-specific training or recalibration might be needed to adapt an existing AIPM to a different healthcare setting^3,5,9,60,68,72^. Statistical updating methods are available for regression-based models^89,90^. For AIPMs outside of this context no specific guidance was found.

Performance analysis by population subgroups or specific problematic use cases is recommended to identify algorithmic bias^6,7,23,24,26,42,61,72,79,91^. Note that such an analysis may be limited by small sample sizes of certain subgroups. The literature advises to discuss and explicitly report any identified sources of algorithmic bias, so that end users know for whom the AIPM’s predictive performance is subpar^8,16^. Many systems will display some unfairness in their outcomes, and therefore a baseline comparison with the algorithmic bias of the current systems may be considered^16^.

### Phase 4. Development of the software application

#### Interoperability

The ability for AIPMs to interoperate with various existing digital infrastructure of hospitals and clinical care centers is essential for their successful integration into healthcare practice. Following existing standards from the industry was recommended as this supports the interoperability of AIPMs^9,18,20,26^ (e.g., ISO/IEC JTC 1/SC 42^92^ or the IEEE 7000-2021^93^). This applies to data coding standards as mentioned in phase 1 of this article, but also to data exchange standards (e.g., FHIR^94^ and the HL7 framework^95^). Such standards provide (among other aspects) guidance on what data formats to use, how they should be exchanged between system components, and reduce the risk that data are accidentally misinterpreted due to slight differences in meaning of variables (semantic interoperability). For wearable devices, following the ISO/IEEE 11073-10418:2014^96^ standard is advised^20^.

Moreover, multiple articles recommend the use of open source or publicly available libraries in the software implementation of the AIPM^20,26^ to increase the accessibility of the AIPM as a whole. The NHS guide to good practice for digital and data-driven health technologies goes as far as to recommend that all new digital health services, including AIPMs, should be made internet-facing from day one (and follow the Representational State Transfer design principles) to promote accessibility and reduce complexity and costs of incorporating them in the digital infrastructure of organizations^20^.

#### Human–AI interaction

A proper design of how end users can interact with the AIPM is crucial for its adoption, and effective and safe use in daily healthcare practice. What constitutes a good design depends on the domain, healthcare setting and intended end users. End users interacting with the AIPM can be healthcare professionals, auditors, or patients (e.g., physicians may need to communicate about the AIPM with patients^18^). Many of the recommendations for human-AI interaction design come from the general human-computer interaction literature and current standards for general medical software design. Recommended standards are ISO 9241-210:2019^97^ for interactive systems and the IEC 62366-1:2015^98^ on application of usability engineering to medical devices^20^. At the software development stage, it has been recommended to include experts in user interface design^8,18^. Designing a good user interface and interaction requires careful consideration of the cognitive load of the end users^10,18,68,99,100^, by showing only relevant information in the right context, and by allowing adjustment of its behavior by end users^99^.

A widely suggested minimum criteria for AIPM user interaction design is that it becomes clear to end users what the AIPM’s intended use is^26,79,87,99^. Providing a model facts label should be provided to the end users is advised, including the system’s technical specifications, statistical working, limitations, fairness criteria and validation, implementation disclaimer, and links to process logs^22,101^.

To arrive at a good design, repeated extensive user experience testing is recommended^11,18^. The AIPM should be evaluated according to how it interfaces with the end user, and how well the AIPM and the user perform together in a typical environment^10,100,102,103^. It was proposed that such evaluations can, for example, be done via reader and user studies^10,102,103^. Tools such as a system usability scale (SUS) have been suggested as a quick and useful way of capturing user feedback^20^.

Careful attention should be paid to inclusiveness and broad usability of the design^20,22,26,62^, for example by considering the digital literacy of the end users^20,22,26^. Multiple sources state that the design should match social norms, and make sure its presentation does not reinforce stereotypes (e.g., regarding a prespecified fairness position or bias impact statement, see phase 2)^22,24,26,32,99^.

Moreover, the AIPM should have built-in mechanisms that protect the end user and patient from potential risks to its safe application (e.g., overconfidence in the AIPMs predictions or automation bias). These mechanisms should detect situations beyond the capabilities of the AIPM^10,99^, and share the confidence in the predictions with the user^10,22,26,99^. Additional information may be required explaining how the confidence level relates to the input data^23,43,61^. It was recommended to carefully consider whether predictions should be presented in a directive fashion (by also proposing decisions), or in an assistive way (e.g., by only showing estimated probabilities)^9,22,40,68,86,87^.

The literature advised that the design should facilitate AIPM interpretability (see also Box 2. and the section on model selection and interpretability in phase 2) and allow end users to visually see the link between the input data and the predicted output^8,10,22,26,32,61,99^ in a comprehensive way^22–24,26,40,42,62^, and encourage giving feedback, correction and refinement about the AIPM’s predictions^99^. Also, the design should enable the patient to request a review of an AIPM-supported decision^63^, and may need to provide the possibility to delete data (depending on local legislation, see phase 1 on Patient privacy)^12,23,36,41^.

#### Facilitating software updating and monitoring

From a user interaction design perspective, it has been recommended that decisions are deterministic (consistently giving the same output for a certain input)^10^, and that updates of or adaptations to the AIPM should happen cautiously^99^. End users should be notified clearly about any changes in the AIPM^26,99^, and AIPM software should have the ability to roll back to previous versions, in case an update results in significant problems^20,66^.

Finally, as monitoring and auditing of AIPMs in practice are widely recommended (covered in more detail in phase 6), the developed software should facilitate this^10,22,26,32,58,62,104^. This means adequate logging and traceability of predictions and decisions is required and the AIPM interface should provide sharing of performance data with end users to enable ongoing monitoring of both individual and aggregated cases, quickly highlighting any significant deviations in performance^10,26,61,66^. Such monitoring options should preferably be customizable by the user^79,99^.

#### Security

The principles of security and privacy by design mandate built-in data and software protection throughout the AIPM lifecycle^12,35,41–43^, which is a central requirement in the GDPR^105^. Cybersecurity standards provide guidance on how to approach this^20,23,26^, for example ANSI/NEMA NH 1-2019^106^, NEN 7510^107^, MDCG 2019-6^108^, ANSI/CAN/UL 2900-1^109^, Medical Device Cybersecurity Working Group on medical device cybersecurity^110^, Food and Drug Administration on cybersecurity^111^, ISO/IEC TS 27110:2021^112^, ISO/IEC 27032:2012^113^, ISO/IEC 27014:2013^114^, and ISO/IEC 27002:2013^115^. This might for example entail an initial risk assessment of vulnerabilities in data and software, including the risk of re-identification^33^, the risk of data loss and manipulation^33,35^, and the risk of adversarial attacks^9,22,23,26,35,43,59^. Techniques that make the AIPM more robust to these vulnerabilities can be implemented, like converting data to less identifiable formats^23^, adding random noise to the data^23,34,41^, federated learning^23,34,41^, saving personal data across different databases^34,35^, and adversarial ML techniques such as model hardening and run-time detection^22,42,43,59^. Code review by an external party and staying up to date on security alerts for code derived from third parties are also recommended^23,35^. All security measures should be tested before full deployment^79^ (also see Software testing). The level of the required security measures will depend on the impact a potential security breach might have on the individuals involved, the type of AI deployed, and the risk management capabilities of the organization^23,27,35,41^. The timeframe within which security updates will become available should be reported^26^.

An incident response plan anticipating a potential security breach is recommended before deployment (also part of western legislation^104,105,116^), describing how incidents will be addressed and who takes responsibility with relevant contact information^23,35,61^. When new software vulnerabilities come to light, they should be documented and reported^32,61^, and so should any changes made to the AIPM in response to an attack after thorough testing^10,23,35,61^.

#### Software testing

AIPM software developers are recommended to follow relevant existing international standards with regard to software testing, such as the IEC 62304:2006^117^, the IEC 82304-1:2016^118^, IEC 62366-1:2015^98^, ISO 14971:2019^119^, Food and Drug Administration principles of software validation^120^, and Food and Drug Administration guidance for off-the-shelf software use in medical devices^121^. Deliberate stress tests like load testing, penetration testing, integration testing and unit testing are important for the verification of the AIPM from a software perspective^10,26,35,46,66,79^. Each different context of use may require separate software testing to ensure reproducibility of results across different situations, computational frameworks, and input data^58,62,88^. These testing requirements depend on the level of reliability needed and the risks posed by the AIPM in healthcare practice^26^. These types of tests are also recommended to assess the effectiveness of the security measures taken and to detect new security vulnerabilities (see Security). They should be repeated regularly to monitor the data and software security during the AIPM lifecycle^23,26,35^.

### Phase 5. Impact assessment of the AIPM with software

#### Feasibility study

An impact assessment is performed to determine the clinical benefit of the AIPM for healthcare practice. It is important to note that a good performance of the AIPM in terms of discrimination and calibration (phases 2 and 3) does not necessarily translate to clinical utility^5,27,72^.

A feasibility study or implementation pilot is recommended preceding an impact study to ensure correct and safe use in healthcare practice^10,18,72^. This type of study consists of repeated live clinical tests in which variation is key to understanding the functionality of the technology and workflow^11,18^. By adhering to the ‘plan, do, study, adjust’ process, adjustments can be made frequently and rapidly to optimize the workflow^11,18^.

The literature advises to clearly define the intended use and intended users in the preparation of both the feasibility and impact study^12,19,64,65^. It is also recommended to report any differences in healthcare setting between the current and previous (validation) studies^68^ and to state the inclusion and exclusion criteria at the level of the participants and input data^28,64,65^. A description of the integration into the trial setting is highly recommended, including onsite and offsite requirements, version number and other technical specifications^28,64,65^, but also the human-AI interaction involved (e.g., assistive versus directive, see phase 4)^46,64^ and the patient treatment strategy associated with the AIPM outcomes^64,65^. It is emphasized that potential interventions included in the patient treatment strategy following from the AIPM decision support should have a solid scientific basis^68^. Stakeholders have preferably given informed approval of the development and clinical application of the AIPM^88^.

#### Risk management

Risk management is highlighted as an important part of the impact assessment, alongside the preparations for a comparative study^28,43^. The literature recommends the identification of potential sources of risk, extreme situations, and failures before the onset of the study^26,57,58^. Determining corresponding safety critical levels and quality checks is advised^26^. Special attention may be paid to accidental misuse and manipulation of the AIPM. Implementers are urged to report errors, failures or near misses occurring during impact assessment and afterwards^24,26,43,61,64,65^. A risk management plan can help to execute the monitoring, reporting and mitigation of risks encountered in healthcare practice^12,16,20,26,28^. This plan can for example describe the roles and responsibilities of the participants^28^, the process for assessing and logging potential risks^12,20,24,26,43,61^, a pathway to report potential risks^12,24,26,43,62^, and the process to address these issues in practice^12,43,62^. Some sources suggest that the assessment should be proportionate to the risk posed by the AIPM^26,43^.

#### Impact study

In terms of the impact study design, a prospective comparative study is recommended^5,8,19,27,56,68,72,86,88^. In a comparative study, the effects on clinical outcomes and decision making are compared for a group exposed to the predictions of the AI versus a non-exposed control group receiving standard care^5,28,68,86,88^. The literature identifies a randomized controlled trial (RCT) as the ideal comparative study design, randomizing patients individually or per cluster^5,9,49,68,86^. However, this may require more patients and might not always be feasible. Alternative designs are stepped-wedge trials^9,19,86^, before-after studies^86^, and observational studies^5,19,56,68,86^. For some applications (like imaging technology), a multiple reader multiple case study design is also possible^46^, in which the effect of the AIPM on decision making is measured by assessing the differences in discrimination (see phases 2 and 3) with and without the tool. Decision Analytical Modeling may give an initial estimate of clinical utility before commencing a full-blown impact study (see phases 2 and 3)^68,86^.

Trial outcomes can differ across domains and applications. The most mentioned trial outcomes consist of clinical outcomes or patient-reported outcomes^5,16,20,68,72,86,88^ followed by cost effectiveness of care^5,16,20,86,88^ and changes in decision making and workflow^5,20,68,86^. Additional trial outcomes are patient experience^20,56,88^, user satisfaction and engagement^88^, and changes in patient (healthy) behavior^88^. It is advised that trial outcomes are also evaluated per clinically relevant user group^12^ or per affected non-user group (also in terms of algorithmic bias)^12,24,91^.

It is recommended that findings are communicated in an understandable and meaningful way to healthcare professionals, but also to administrators and policymakers^57^. AIPM-specific guidelines have been developed as extensions to the CONSORT and SPIRIT guidelines for reporting on clinical trials and their protocols respectively^64,65^. Peer-reviewed open access publication may increase trust and facilitate adoption of the AIPM in a wider clinical community^9^.

### Phase 6. Implementation and use in daily healthcare practice

#### Clinical implementation

Clinical implementation consists of all the steps that are necessary to deploy the AIPM in the healthcare environment outside of the clinical trial setting (see phase 5). The literature strongly recommends to state the necessary conditions for deployment before proceeding with the implementation^11,19,20,26,87^. For example, the AIPM system might require dedicated and locally available hardware^8^.

Although not always feasible, the integration of an AIPM directly into the existing medical workflow is preferred^8,19,59,68^. This could for example involve direct integration into the EHR. Moreover, the user is urged to explicitly disclose what part of decision making might be affected by AIPM predictions^24,26,42,62,63,87^.

To further facilitate the implementation and consecutive monitoring, the literature recommends automatic AIPM deployment (moving software from testing to production environments with automated processes) and the facilitation of shadow deployment^66,91^, which enables prospective local validation (see phase 3) of new versions and updates^19^. Enabling the automatic roll-back for production models is also advised to address real-time operating risks (see phase 4)^66^. Moreover, a procedure to safely abort an operation is highly recommended when the system should stop being used due to a security breach or safety risk^23,26,62,79^. Comparable to the feasibility study of phase 5, pilot studies are recommended to examine the potential pitfalls during implementation, considering both software and hardware issues^10,18,72^.

Lastly, Institutions and implementers are encouraged to disclose their innovation pathway, including the routes to commercialization^16^. The risks, investments, roles, and responsibilities of the different parties may inform the allocation of benefits in a commercial arrangement^16,20^. Albeit sparse,^88^ provide good guidance on performing economic impact analysis.

#### Maintenance and updating

Although maintenance is essential to AIPMs (and their software) that are highly dependable on changes in the external world, little guidance can be found on this topic. Developers are recommended to regularly update their AIPMs over time to improve the AIPM’s predictive performance as new improvements become available and to mitigate dataset shift^10,19,23^. It is advised to pay special attention to the safe and automatic updating of mature systems involving many configurations for many similar models^71^. Note that updating the AIPM may involve recertification. The USA Food and Drug Administration is currently working on a framework that allows for repeated updating of an AIPM without repeated recertification through a change control plan^122^.

#### Education

Education involves the training of end users in the correct use of the AIPM. The literature recommends the general education of end users, often healthcare professionals, on the probabilistic nature^22–24,42^ and the limitations of AIPMs^22,42^. This may involve the development of a general AI curriculum for medical students and healthcare professionals.

Application specific training is also advised. The end user may for example be educated on the underlying assumptions of the AIPM^58,68^, its legal framework^26^, benefits^20,26,58^, risks and (technical) limitations^15,22,26,58,62^. Providing the end user with examples of incorrectly classified cases could help in creating an understanding of the strengths and limitations of the AIPM^14^. Moreover, it is recommended to regularly repeat the training on the correct use of the AIPM^12,15,26,58,62^ and the appropriate response to security breaches^23,35^. For example, end users may be made aware of the possibility of automation bias and trained to maintain vigilance^22,26,57,87,91^.

When the end user (healthcare professional) and AIPM subject (patient) are different people, as is often the case for AIPMs in healthcare, the literature recommends to train the healthcare professional to explain one’s AIPM-supported decisions to their patient^22^.

#### Monitoring and auditing

Monitoring refers to the post-deployment evaluation of the behavior of an AIPM throughout its lifecycle^10,23,26,27,57,62,64,66,72,80,91^. It is performed by the developer and implementers at the implementation site. Auditing refers to periodic quality control checks of the AIPM (and all of its monitoring aspects) performed by an independent third party^26,58,62,91^. Among other things, It will aid the detection of failures and near misses and through this strengthen the risk management and security of an AIPM^35,58^.

Several aspects of AIPM functioning can be monitored as identified in the literature. These may for example consist of predictive performance and other model outputs^9,10,26,57,63,79,80^, distribution of predicted versus observed labels^71^, reliability and reproducibility^10,26,62^, types and severity of errors^57^, changes in risk^80^, quality of the input data^26,57,63,71,88^, quality of the label^91^, case-mix factors^72,91^, accessibility and integration of the model^57^, use of the AIPM recommendations^57,63,88^, user satisfaction and user feedback^9,10,57,79,88^, and (clinical) outcomes^26,57,80,88^.

Several monitoring aspects are highlighted in the literature that deserve additional scrutiny. The monitoring of the fairness of an AIPM throughout its lifecycle is often mentioned^9,12,20,23,24,26,63^, for example by recording false positive and false negative prediction rates sliced across different subgroups^25,26,79,91^. Second, the monitoring of dataset shift is also repeatedly mentioned in the literature^5,10,22,72,79,91^. Dataset shift is a change in the composition of the input data caused by changes in clinical or operational practices over time that can lead to the deterioration of AIPM performance. It can for example be measured by an increase in classification errors over time^23^. It can be mitigated by retraining or updating the AIPM^72^. One last aspect is the monitoring of feedback loops^26^. They originate when an AIPM is modeled on care delivery features that in turn might be affected by the outcomes of an AIPM.

It is advised to develop integrated mechanisms to facilitate real-time monitoring available at the start of implementation^16,71^. Implementers are encouraged to clearly define the context and boundaries within which the monitoring is to be performed^57^. Specifying the type of oversight is also recommended, e.g., human-in-the-loop, human-on-the-loop, or human-in-command^26^. Some sources suggest the frequency of the monitoring should be proportional to the AIPM’s risks^22,23,91^: the higher the risk to the welfare of the patient, the higher the monitoring frequency should be. One source suggests frequent monitoring may be less important for AIPMs solely based on causal mechanisms as they are less likely to change over time^27^.

In terms of auditing, the literature recommends the installation of a comprehensive auditability framework^10,22,58^ and an audit trail^25,46,62^, in which the AIPM’s predictions, model version, input data, and use practices are methodologically logged and made available to interested third parties^22,26,32,35,58,61,62,66,91^.

Implementers are advised to define mitigation pathways as part of the monitoring and auditing plan to deal with incidents^22,35,71,79^. This may for example involve the regular reporting on failures and near misses and the organization of meetings to discuss incidents^58^. Moreover, the literature states that mitigation could and sometimes should lead to a change in the AIPM’s design or use practices, for example an adjustment in the instructions for use, a re-evaluating of the stakeholder impact assessment, or a model update^22,72,80^.

### Current gaps and future perspectives

We identified several important aspects of the AIPM development, evaluation and implementation cycle for which clear guidance was missing in the literature. First, guidance is lacking on the requirements to be fulfilled during the assessment of the medical problem and context. In other words, what aspects of a medical or healthcare problem and setting make the introduction of an AIPM likely to result in better patient care, and when are conditions sufficient to initiate AIPM development? Guidance is also missing on the a priori estimation of a minimum sample size for AIPM development for semi-supervised approaches, and for certain commonly used groups of ML modeling techniques such as decision trees (e.g., random forests) and deep learning (e.g., convolutional neural networks).

Across all phases, several methodologies and quality criteria were identified to address ethical issues such as algorithmic bias, privacy preservation, and interpretable AI. However, the relevance of these issues for different healthcare domains might differ and so will the preferred definitions, metrics, and techniques to describe and mitigate them. As domain specific guidelines were not the primary focus of this investigation, we cannot with certainty comment on the general absence of such guidelines. Nevertheless, we would advise individual healthcare domains to scrutinize the currently available guidance and, when necessary, address these ethical issues across the AIPM development, evaluation and implementation cycle for their respective settings.

Another aspect for which guidance was limited, is the combination of different data sources (e.g., from different registries and collection sites), and data modalities (e.g. imaging data, electrophysiological data, and lab results) for AIPM development. Although methodological studies exist for various combinations, further research on best practices is needed. Also, current guidance is primarily focused on binary outcomes (e.g., mortality), and guidance is missing on other outcome types (e.g., multinomial, ordinal, hierarchical or sequential outcomes).

Although many standards exist for software security, it is unclear whether they suffice to address cyberattacks particularly geared at AIPMs. Experience with AIPM security in practice and experimentation with the insulation of AIPMs against different types of cyberattack in preclinical settings will help to clarify this. Also, more guidance on the unique aspects of AIPM-specific human-AI interaction design is needed. This will for example entail the presentation of and interaction with probabilistic outcomes and the impact of model interpretability on end users.

Much more guidance is needed addressing how to integrate the AIPM into the current healthcare or clinical workflow. More guidance is also required specifying what design and execution of the feasibility and impact studies are needed, and how to report such studies.

Moreover, guidance is needed regarding the assessment of the cost effectiveness of AIPMs. AIPMs differ from other health technologies and are likely to affect healthcare differently, which should be reflected in their cost effectiveness assessments (as was done for the guidance on impact studies).

We described recommendations regarding the responsibilities of different parties (developers, end users, organizations) involved with AIPM development and deployment as described in the identified literature (e.g., risk assessment, incident reporting, patient privacy). However, more work is needed addressing the proper distribution of accountability across all involved parties, which may in turn inform institutional governance.

Lastly, guidance is needed on (long-term) maintenance aspects, on dataset shift (and how to mitigate it), and on the frequency and necessity of local validation, recalibration (updating), and retraining. As more and more AIPMs will be implemented into healthcare practice in the coming years, this practical experience can be used to inform these aspects.

## Discussion

This scoping review provides an easy-to-use overview and summary of the currently available actionable guidelines and quality criteria driven by the six phases of the AIPM development, evaluation, and implementation cycle: (1) data preparation, (2) AIPM development, (3) AIPM validation, (4) software development, (5) AIPM impact assessment, and (6) AIPM implementation into daily healthcare practice. Guidance was structured in specific topics and mapped to the different phases and we provided an overview of the current gaps in this guidance.

To appreciate our scoping review and suggested framework of six phases several issues need to be addressed. First, our definitions of ‘actionable’ guidance as an inclusion criterion and the defined six phases are somewhat arbitrary and mainly informed by vast experience with and guidance on developing, evaluating, and implementing prediction models in healthcare. Individual AIPM applications may deviate from the structure presented here. Nevertheless, we believe the phases and their associated topics will translate to most AIPM projects and are in agreement with other phases formulated in the literature^4,5,8,22^. Also, the structure provided by the six phases, and our focus on actionability form two strengths of this scoping review and produce a comprehensible and easy-to-use overview of practical recommendations for those involved in the AIPM development, evaluation and implementation cycle. This sets our review apart from other work that was previously undertaken (e.g.,^32,123,124^).

Second, the literature databases and sources we used mostly contain scientific literature and only English documents were included in the final search (translations were also considered). This may have biased our results towards academic sources and English-speaking countries of origin. To combat this, we identified additional gray literature through consultation with AI experts and a thorough screening of citations in the included literature. As a result, a substantial number of our included sources can be considered gray literature. Moreover, due to our extensive search, the current summary of available guidelines and quality criteria is comprehensive.

Lastly, the expert group consulted was a convenience sample, resulting in experts predominantly working in the Netherlands. Diversity was obtained by inviting experts with different occupations (e.g., healthcare professionals, data scientists, statisticians, engineers), from different healthcare domains (e.g., radiology, internal medicine, intensive care, primary care, family medicine), and from both academia and industry.

In conclusion, a substantial number of studies provide guidelines and quality criteria pertaining to the AIPM development, evaluation, and implementation cycle, which can be grouped into six well-defined phases. While the opportunities of AIPMs in healthcare are undeniable, the growing interest in these techniques requires careful quality and applicability assessment to guarantee their safety and (cost-)effectiveness before they are used and disseminated in healthcare. This review can serve as the basis for a structured quality assessment framework. Several gaps in the literature were identified where more research is needed. Additional domain and technology specific studies may be necessary and more practical experience with implementing AIPMs is needed to inform further guidance.

## Supplementary information

Supplementary Information

## Data Availability

The authors declare that all data supporting the findings of this study are available within the paper and its supplementary information files.
