# Supplementary material for: Guidelines and quality criteria for artificial intelligence-based prediction models in healthcare: a scoping review
Source: NPJ Digit Med. 2022 Jan 10;5:2. doi: 10.1038/s41746-021-00549-7 (PMC8748878; doi:10.1038/s41746-021-00549-7)
Supplement: Supplementary file 1 — Supplementary Information [file 41746_2021_549_MOESM1_ESM.pdf]

Supplementary information

Supplementary Table 1. The search query

Database	Search string
PubMed	(("data cleaning" OR "data preparation" OR "preprocessing" OR "pre-processing" OR "design" OR "missing data" OR "outlier detection" OR "data harmonization" OR "de-identification" OR "anonymization" OR "predictor selection " OR "feature selection" OR "feature extraction" OR "selection bias" OR "annotation" OR "sample size" OR "data privacy" OR "model development" OR "model architectures" OR "explainability" OR "hyper-parameter" OR "hyperparameter" OR "model training" OR "model fitting" OR "optimization" OR "model updating" OR "parameter-sharing" OR "distant supervision" OR "weak supervision" OR "interpretability" OR "interpretable" OR "evaluation" OR "calibration" OR "discrimination" OR "evaluation metric" OR "evaluation measure" OR "model accuracy" OR "risk of bias" OR "prediction performance" OR "generalization error" OR "prediction error" OR "validation" OR "net-benefit" OR "precision" OR "software" OR "software quality assurance" OR "SQA" OR "software quality control" OR "SQC" OR "software as a medical device" OR "SaMD" OR "software compliance" OR "anti-patterns" OR "design patterns" OR "software architecture" OR "front-end design" OR "presentation layer" OR "software testing" OR "software security" OR "Software as a service" OR "SaaS" OR "miminum viable product" OR "impact assessment" OR "outcome assessment" OR "validation" OR "clinical performance" OR "clinical investigation" OR "external validation" OR "RCT" OR "randomized controlled trial" OR "randomized clinical trial" OR "random control trial" OR "technology assessment" OR "HTA" OR "clinical impact" OR "pilot study" OR "clinical benefit" OR "clinical evaluation" OR "cost-effectiveness" OR "generalizability" OR "explainability" OR "clinical benchmarking" OR "study design" OR "fairness" OR "bias" OR "qualitative evaluation" OR "implementation" OR "scalability" OR "integration" OR "calibration" OR "transfer learning" OR "usability" OR "patient satisfaction" OR "satisfaction" OR "interoperability" OR "user friendly" OR "ethics" OR "ethical" OR "jurisprudence" OR "legislation" OR "legal" OR "law" OR "diffusion" OR "application" OR "dissemination" OR "real-world performance" OR "real world performance" OR "monitoring" OR "clinical practice" OR "education") AND ("artificial

	intelligence"[Title] OR "machine intelligence"[Title] OR "machine learning"[Title] OR "deep learning"[Title] OR "prediction model"[Title] OR "neural network"[Title] OR "support vector machines"[Title] OR "natural language processing"[Title] OR "computer vision"[Title] OR "supervised learning"[Title] OR "unsupervised learning"[Title] OR "reinforcement learning"[Title] OR "statistical learning"[Title] OR "computational intelligence"[Title] OR "computer reasoning"[Title] OR "AI"[Title] OR "computer heuristics"[Title] OR "expert systems"[Title])) AND ("recommendations"[Title] OR "challenges"[Title] OR "guideline"[Title] OR "a guide"[Title] OR "guidelines"[Title] OR "practice guideline"[Title] OR "practice guidelines"[Title] OR "quality norm"[Title] OR "quality of care"[Title] OR "quality criteria"[Title] OR "quality instrument"[Title] OR "quality of health care"[Title] OR "healthcare quality"[Title] OR "quality improvement"[Title] OR "quality indicator"[Title] OR "quality indicators"[Title] OR "total quality management"[Title] OR "best practice"[Title] OR "code of conduct"[Title] OR "reporting standard"[Title] OR "good machine learning practice"[Title] OR "best practices"[Title] OR "framework"[Title] OR "guidance"[Title] OR "strategies for"[Title] OR "statement"[Title])
Web of Science	TS=(("guideline" OR "a guide" OR "guidelines" OR "practice guideline" OR "practice guidelines" OR "quality norm" OR "quality of care" OR "quality criteria" OR "quality instrument" OR "quality of health care" OR "healthcare quality" OR "quality improvement" OR "quality indicator" OR "quality indicators" OR "total quality management" OR "best practice" OR "code of conduct" OR "reporting standard" OR "good machine learning practice" OR "best practices" OR "framework" OR "guidance" OR "strategies for" OR "statement" OR "recommendations" OR "challenges") AND ("artificial intelligence" OR "machine intelligence" OR "machine learning" OR "deep learning" OR "prediction model" OR "neural network" OR "support vector machines" OR "natural language processing" OR "computer vision" OR "supervised learning" OR "unsupervised learning" OR "reinforcement learning" OR "statistical learning" OR "computational intelligence" OR "computer reasoning" OR "AI" OR "computer heuristics" OR "expert systems")) AND AB=(("data cleaning" OR "data preparation" OR "preprocessing" OR "pre-processing" OR "design" OR "missing data" OR "outlier detection" OR "data harmonization" OR "de-identification" OR "anonymization" OR "predictor selection " OR "feature selection" OR "feature extraction" OR "selection bias" OR "annotation" OR

	"sample size" OR "data privacy" OR "model development" OR "model architectures" OR "explainability" OR "hyper-parameter" OR "hyperparameter" OR "model training" OR "model fitting" OR "optimization" OR "model updating" OR "parameter-sharing" OR "distant supervision" OR "weak supervision" OR "interpretability" OR "interpretable" OR "evaluation" OR "calibration" OR "discrimination" OR "evaluation metric" OR "evaluation measure" OR "model accuracy" OR "risk of bias" OR "prediction performance" OR "generalization error" OR "prediction error" OR "validation" OR "net-benefit" OR "precision" OR "software" OR "software quality assurance" OR "SQA" OR "software quality control" OR "SQC" OR "software as a medical device" OR "SaMD" OR "software compliance" OR "anti-patterns" OR "design patterns" OR "software architecture" OR "front-end design" OR "presentation layer" OR "software testing" OR "software security" OR "Software as a service" OR "SaaS" OR "minimum viable product" OR "impact assessment" OR "outcome assessment" OR "validation" OR "clinical performance" OR "clinical investigation" OR "external validation" OR "RCT" OR "randomized controlled trial" OR "randomized clinical trial" OR "random control trial" OR "technology assessment" OR "HTA" OR "clinical impact" OR "pilot study" OR "clinical benefit" OR "clinical evaluation" OR "cost-effectiveness" OR "generalizability" OR "explainability" OR "clinical benchmarking" OR "study design" OR "fairness" OR "bias" OR "qualitative evaluation" OR "implementation" OR "scalability" OR "integration" OR "calibration" OR "transfer learning" OR "usability" OR "patient satisfaction" OR "satisfaction" OR "interoperability" OR "user friendly" OR "ethics" OR "ethical" OR "jurisprudence" OR "legislation" OR "legal" OR "law" OR "diffusion" OR "application" OR "dissemination" OR "real-world performance" OR "real world performance" OR "monitoring" OR "clinical practice" OR "education") AND (Healthcare domain))
ACM Digital Library	[[Publication Title: "recommendations"] OR [Publication Title: "challenges"] OR [Publication Title: "guideline"] OR [Publication Title: "a guide"] OR [Publication Title: "guidelines"] OR [Publication Title: "practice guideline"] OR [Publication Title: "practice guidelines"] OR [Publication Title: "quality norm"] OR [Publication Title: "quality of care"] OR [Publication Title: "quality criteria"] OR [Publication Title: "quality instrument"] OR [Publication Title: "quality of health care"] OR [Publication Title: "healthcare quality"] OR [Publication Title: "quality improvement"] OR [Publication Title: "quality indicator"] OR [Publication Title: "quality indicators"] OR [Publication Title: "total

	quality management"] OR [Publication Title: "best practice"] OR [Publication Title: "code of conduct"] OR [Publication Title: "reporting standard"] OR [Publication Title: "good machine learning practice"] OR [Publication Title: "best practices"] OR [Publication Title: "framework"] OR [Publication Title: "guidance"] OR [Publication Title: "strategies for"] OR [Publication Title: "statement"]] AND [[Publication Title: "artificial intelligence"] OR [Publication Title: "machine intelligence"] OR [Publication Title: "machine learning"] OR [Publication Title: "deep learning"] OR [Publication Title: "prediction model"] OR [Publication Title: "neural network"] OR [Publication Title: "support vector machines"] OR [Publication Title: "natural language processing"] OR [Publication Title: "computer vision"] OR [Publication Title: "supervised learning"] OR [Publication Title: "unsupervised learning"] OR [Publication Title: "reinforcement learning"] OR [Publication Title: "statistical learning"] OR [Publication Title: "computational intelligence"] OR [Publication Title: "computer reasoning"] OR [Publication Title: "ai"] OR [Publication Title: "computer heuristics"] OR [Publication Title: "expert systems"]] AND [[Full Text: "data cleaning"] OR [Full Text: "data preparation"] OR [Full Text: "preprocessing"] OR [Full Text: "pre-processing"] OR [Full Text: "design"] OR [Full Text: "missing data"] OR [Full Text: "outlier detection"] OR [Full Text: "data harmonization"] OR [Full Text: "de-identification"] OR [Full Text: "anonymization"] OR [Full Text: "predictor selection "] OR [Full Text: "feature selection"] OR [Full Text: "feature extraction"] OR [Full Text: "selection bias"] OR [Full Text: "annotation"] OR [Full Text: "sample size"] OR [Full Text: "data privacy"] OR [Full Text: "model development"] OR [Full Text: "model architectures"] OR [Full Text: "explainability"] OR [Full Text: "hyper-parameter"] OR [Full Text: "hyperparameter"] OR [Full Text: "model training"] OR [Full Text: "model fitting"] OR [Full Text: "optimization"] OR [Full Text: "model updating"] OR [Full Text: "parameter-sharing"] OR [Full Text: "distant supervision"] OR [Full Text: "weak supervision"] OR [Full Text: "interpretability"] OR [Full Text: "interpretable"] OR [Full Text: "evaluation"] OR [Full Text: "calibration"] OR [Full Text: "discrimination"] OR [Full Text: "evaluation metric"] OR [Full Text: "evaluation measure"] OR [Full Text: "model accuracy"] OR [Full Text: "risk of bias"] OR [Full Text: "prediction performance"] OR [Full Text: "generalization error"] OR [Full Text: "prediction error"] OR [Full Text: "validation"] OR [Full Text: "net-benefit"] OR [Full Text: "precision"] OR [Full Text: "software"] OR [Full Text: "software quality assurance"] OR [Full Text: "sqa"] OR [Full Text:

	"software quality control"] OR [Full Text: "sqc"] OR [Full Text: "software as a medical device"] OR [Full Text: "samd"] OR [Full Text: "software compliance"] OR [Full Text: "anti-patterns"] OR [Full Text: "design patterns"] OR [Full Text: "software architecture"] OR [Full Text: "front-end design"] OR [Full Text: "presentation layer"] OR [Full Text: "software testing"] OR [Full Text: "software security"] OR [Full Text: "software as a service"] OR [Full Text: "saas"] OR [Full Text: "miminum viable product"] OR [Full Text: "impact assessment"] OR [Full Text: "outcome assessment"] OR [Full Text: "validation"] OR [Full Text: "clinical performance"] OR [Full Text: "clinical investigation"] OR [Full Text: "external validation"] OR [Full Text: "rct"] OR [Full Text: "randomized controlled trial"] OR [Full Text: "randomized clinical trial"] OR [Full Text: "random control trial"] OR [Full Text: "technology assessment"] OR [Full Text: "hta"] OR [Full Text: "clinical impact"] OR [Full Text: "pilot study"] OR [Full Text: "clinical benefit"] OR [Full Text: "clinical evaluation"] OR [Full Text: "cost-effectiveness"] OR [Full Text: "generalizability"] OR [Full Text: "explainability"] OR [Full Text: "clinical benchmarking"] OR [Full Text: "study design"] OR [Full Text: "fairness"] OR [Full Text: "bias"] OR [Full Text: "qualitative evaluation"] OR [Full Text: "implementation"] OR [Full Text: "scalability"] OR [Full Text: "integration"] OR [Full Text: "calibration"] OR [Full Text: "transfer learning"] OR [Full Text: "usability"] OR [Full Text: "patient satisfaction"] OR [Full Text: "satisfaction"] OR [Full Text: "interoperability"] OR [Full Text: "user friendly"] OR [Full Text: "ethics"] OR [Full Text: "ethical"] OR [Full Text: "jurisprudence"] OR [Full Text: "legislation"] OR [Full Text: "legal"] OR [Full Text: "law"] OR [Full Text: "diffusion"] OR [Full Text: "application"] OR [Full Text: "dissemination"] OR [Full Text: "real-world performance"] OR [Full Text: "real world performance"] OR [Full Text: "monitoring"] OR [Full Text: "clinical practice"] OR [Full Text: "education"]] AND [Publication Date: (01/01/2000 TO *)]

Supplementary Table 2. The filled PRISMA-ScR checklist (Systematic reviews and Meta-Analyses extension for Scoping Reviews).

SECTION	ITEM	PRISMA-ScR CHECKLIST ITEM	REPORTED IN
**TITLE**			
Title	1	Identify the report as a scoping review.	Title
**ABSTRACT**			
Structured summary	2	Provide a structured summary that includes (as applicable): background, objectives, eligibility criteria, sources of evidence, charting methods, results, and conclusions that relate to the review questions and objectives.	Abstract
**INTRODUCTION**			
Rationale	3	Describe the rationale for the review in the context of what is already known. Explain why the review questions/objectives lend themselves to a scoping review approach.	Introduction
Objectives	4	Provide an explicit statement of the questions and objectives being addressed with reference to their key elements (e.g., population or participants, concepts, and context) or other relevant key elements used to conceptualize the review questions and/or objectives.	Introduction
**METHODS**			
Protocol and registration	5	Indicate whether a review protocol exists; state if and where it can be accessed (e.g., a Web address); and if available, provide registration information, including the registration number.	No protocol was registered in advance.
Eligibility criteria	6	Specify characteristics of the sources of evidence used as eligibility criteria (e.g., years considered, language, and publication status), and provide a rationale.	Methods

Information sources*	7	Describe all information sources in the search (e.g., databases with dates of coverage and contact with authors to identify additional sources), as well as the date the most recent search was executed.	Methods<br>Figure 1<br>Supplementary Table 3
Search	8	Present the full electronic search strategy for at least 1 database, including any limits used, such that it could be repeated.	Methods<br>Figure 1<br>Supplementary Table 1
Selection of sources of evidence†	9	State the process for selecting sources of evidence (e.g., screening and eligibility) included in the scoping review.	Methods
Data charting process‡	10	Describe the methods of charting data from the included sources of evidence (e.g., calibrated forms or forms that have been tested by the team before their use, and whether data charting was done independently or in duplicate) and any processes for obtaining and confirming data from investigators.	Methods<br>Supplementary Table 4
Data items	11	List and define all variables for which data were sought and any assumptions and simplifications made.	Methods<br>Supplementary Table 4
Critical appraisal of individual sources of evidence§	12	If done, provide a rationale for conducting a critical appraisal of included sources of evidence; describe the methods used and how this information was used in any data synthesis (if appropriate).	N.A.
Synthesis of results	13	Describe the methods of handling and summarizing the data that were charted.	Methods<br>Supplementary Table 4
**RESULTS**			
Selection of sources of evidence	14	Give numbers of sources of evidence screened, assessed for eligibility, and included in the review, with reasons for exclusions at each stage, ideally using a flow diagram.	Results<br>Figure 1
Characteristics of sources of evidence	15	For each source of evidence, present characteristics for which data were charted and provide the citations.	Supplementary Table 6 and 7

Critical appraisal within sources of evidence	16	If done, present data on critical appraisal of included sources of evidence (see item 12).	N.A.
Results of individual sources of evidence	17	For each included source of evidence, present the relevant data that were charted that relate to the review questions and objectives.	Supplementary Table 6 and 7
Synthesis of results	18	Summarize and/or present the charting results as they relate to the review questions and objectives.	Results<br>Supplementary Table 5<br>Box 2
**DISCUSSION**			
Summary of evidence	19	Summarize the main results (including an overview of concepts, themes, and types of evidence available), link to the review questions and objectives, and consider the relevance to key groups.	Discussion
Limitations	20	Discuss the limitations of the scoping review process.	Discussion
Conclusions	21	Provide a general interpretation of the results with respect to the review questions and objectives, as well as potential implications and/or next steps.	Discussion
**FUNDING**			
Funding	22	Describe sources of funding for the included sources of evidence, as well as sources of funding for the scoping review. Describe the role of the funders of the scoping review.	Acknowledgements

JBI = Joanna Briggs Institute; PRISMA-ScR = Preferred Reporting Items for Systematic reviews and Meta-Analyses extension for Scoping Reviews.
* Where *sources of evidence* (see second footnote) are compiled from, such as bibliographic databases, social media platforms, and Web sites.
† A more inclusive/heterogeneous term used to account for the different types of evidence or data sources (e.g., quantitative and/or qualitative research, expert opinion, and policy documents) that may be eligible in a scoping review as opposed to only studies. This is not to be confused with *information sources* (see first footnote).
‡ The frameworks by Arksey and O'Malley (6) and Levac and colleagues (7) and the JBI guidance (4, 5) refer to the process of data extraction in a scoping review as data charting.
§ The process of systematically examining research evidence to assess its validity, results, and relevance before using it to inform a decision. This term is used for items 12 and 19 instead of "risk of bias" (which is more applicable to systematic reviews of interventions) to include and acknowledge the various sources of evidence that may be used in a scoping review (e.g., quantitative and/or qualitative research, expert opinion, and policy document).

From: Tricco AC, Lillie E, Zarin W, O'Brien KK, Colquhoun H, Levac D, et al. PRISMA Extension for Scoping Reviews (PRISMAScR): Checklist and Explanation. Ann Intern Med. 2018;169:467–473. doi: 10.7326/M18-0850.

Supplementary Table 3. The consulted experts

Name	Affiliation	Expertise
Maarten de Rijke	University of Amsterdam	Artificial intelligence
Evangelos Kanoulas	University of Amsterdam	Machine learning and statistics
Floor van Leeuwen	Quantib	Medical device regulation
Daniel Oberski	Utrecht University	Machine learning and statistics
Wiro Niessen	Erasmus MC, University Medical Center Rotterdam & Delft University of Technology	Medical image processing
Giovanni Cina	Pacmed	Artificial intelligence
Rene Aarnink	Philips	Artificial intelligence
Anonymous	-	Medical device regulation
Bart-Jan Verhoeff	Expertisecentrum Zorgalgoritmen	Clinical software development
Bart Geerts	Healthplus.ai & Spaarne Gasthuis	Clinical AI implementation
Egge van der Poel	Erasmus Medical Center	Personalized healthcare
Stephan Romeijn	Leiden University Medical Center	Clinical AI implementation
Martijn Bauer	Leiden University Medical Center	Clinical AI implementation
André Dekker	Maastricht University	Clinical data science

Supplementary Table 4. The mapping from the initially used more fine-grained topics and terms identified in the literature, to more coarse-grained topics used in the review's outline, and their distribution over the different phases from Box 1.

Keywords	Topic	Phase
Problem definition; analysis of the status quo; background study; specification of the clinical setting; identification of stakeholders; the prediction task; motivation; clinical context; clinical workflow; clinical baseline; clinical setting; clinical question	Medical problem and context	Phase 1
GDPR; privacy; informed consent; data governance; de-identification; lawful basis; data minimization; compliance; ethics	Patient privacy	
Sample size	Sample size	
Representative data; study population; spectrum bias	Representativeness	
Missing data; measurement error; data quality; outliers; inter-annotator agreement; labeling; annotation; ground truth quality; data cleaning; data collection; reference standard; outcome measures; reference test	Data quality	
Data cleaning; data preparation; outlier detection; imputation; data wrangling; data fusion; integrating data; fusion; coding predictors	Data preprocessing	
Data standards; interoperability	Data coding standards	
Interpretability; model selection; deep learning; federated learning; model specification; statistical models	Model selection and interpretability	Phase 2
Parameter tuning; hyperparameters; nested cross-validation; development; training; train-test split	Training the AIPM	
Overfitting; dimensionality reduction; feature selection; class imbalance; clustering; regularization; predictor selection	Measures to reduce risk of overfitting	
Fairness; discriminatory bias; equality; algorithmic bias	Measures to identify and prevent discriminatory bias	
Validation; evaluation; metrics; cross-validation; train-test split; evaluation metrics; calibration; discrimination; internal validation	Internal validation	
Reporting; code sharing	Transparency of the modelling process	
Validation; evaluation; metrics; comparing models; evaluation metrics; external validation; prospective vs. retrospective; calibration; discrimination; benchmarking; temporal validation	Validation of the AIPM	Phase 3
Generalizability	Generalizability	

Interoperability; open source; data standards; software design	Interoperability	Phase 4
Human-AI interaction; usability; human-machine interaction; design; user interface; HCI	Human-AI interaction	
Logging; software updating; facilitating monitoring	Facilitating software updating and monitoring	
Security; adversarial attack; model inversion attack	Security	
Security; risks; software design; coding; unit testing; software testing	Software testing	
Feasibility study; clinical utility; patient treatment strategy	Feasibility study	Phase 5
Effectiveness; clinical outcome; impact assessment; clinical impact assessment; RCT; economic impact assessment	Impact study	
Risks; risk management	Risk management	
Implementation; patient-physician relation; integration into clinical workflow; clinical implementation; trust	Clinical implementation	Phase 6
Hardware; maintenance	Maintenance	
Education	Education	
Auditing; data drift; monitoring; concept drift; performance over time; third-party evaluation;	Monitoring and auditing	
Fairness; algorithmic bias; bias; ethics	Algorithmic bias & fairness	Overarching
Reporting; open source; code sharing; transparency; trust	Transparency & openness	
Explainability; saliency mapping; trust	Interpretability	
Accountability; stakeholders; multidisciplinary team	Development team, end users, and stakeholders	
Security; safety; model inversion attack; adversarial attack; trust	Security	
Risks; safety; risk management	Risks	

Supplementary Table 5. Index on where phase-overarching topics are discussed in the article's summary of the found guidance.

	Topic	Algorithmic bias and fairness	Transparency and openness	Interpretability	Team members, end users, and stakeholders	Security	Risks
**Phase 1**	Medical problem and context	✔	✔	.	✔	.	✔
	Patient privacy	.	✔	.	✔	✔	.
	Sample size	.	✔	.	.	.	.
	Representativeness	✔	✔	.	.	.	.
	Data quality	✔	✔	.	✔	.	✔
	Data preprocessing	.	✔	.	✔	.	✔
	Data coding standards	.	.	.	.	.	.
**Phase 2**	Model selection and interpretability	✔	✔	✔	.	✔	✔
	Training the AIPM	.	✔	.	.	.	.
	Internal validation	.	✔	.	.	.	✔
	Measures to reduce risk of overfitting	.	✔	.	✔	.	✔
	Measures to identify and prevent algorithmic bias	✔	✔	✔	✔	.	.
	Transparency of the modelling process	.	✔	.	.	.	.
**Phase 3**	Validation of the AIPM	.	.	.	✔	.	✔
	Generalizability	✔	✔	.	.	.	✔
**Phase 4**	Interoperability	.	✔	.	.	.	✔
	Human-AI interaction	✔	✔	✔	✔	.	✔

	Facilitating software updating and monitoring	.	✔	.	.	.	✔
	Security	.	✔	.	✔	✔	✔
	Software testing	.	.	.	.	✔	✔
**Phase 5**	Feasibility study	.	✔	.	✔	.	.
	Risk management	.	✔	.	.	.	✔
	Impact study	✔	✔	.	.	.	.
**Phase 6**	Clinical implementation	.	✔	.	.	✔	✔
	Maintenance and updating	.	.	.	.	.	✔
	Education	.	✔	.	✔	✔	✔
	Monitoring and auditing	✔	✔	.	✔	✔	✔

Supplementary Table 6. References identified per topic

	Topic	References
Phase 1	Medical problem and context	Bossuyt et al. 2003, Nykänen et al. 2011, Food and Drug Administration 2012, Steyerberg and Vergouwe 2014, Collins et al. 2015, Luo et al. 2016, Global Future Council on Human Rights 2018, Machine Intelligence Garage's Ethics Committee 2018, Matheny et al. 2019, Cearns et al. 2019, Leslie 2019, Ray et al. 2019, Turner Lee et al. 2019, Wiens et al. 2019, High-Level Expert Group on Artificial Intelligence 2020, Information Commissioner's Office 2020a, Mongan et al. 2020, Norgeot et al. 2020, Stevens et al. 2020, Vollmer 2020, Larson et al. 2021, Smith et al. 2021, UK Department of Health & Social Care 2021, Weikert et al. 2021
	Patient privacy	Datatilsynet 2017, Datatilsynet 2018, Machine Intelligence Garage's Ethics Committee 2018, Benjamins et al. 2019, Arrieta et al. 2020, Gebru et al. 2020, High-Level Expert Group on Artificial Intelligence 2020, Information Commissioner's Office 2020a, Information Commissioner's Office 2020b, Liaw et al. 2020, Mongan et al. 2020, Rodríguez et al. 2020, Sartor and Lagioia 2020, Ryan and Stahl 2021, UK Department of Health & Social Care 2021, Weikert et al. 2021
	Sample size	Bhaskar et al. 2006, Steyerberg 2009, Food and Drug Administration 2012, Collins et al. 2015, Luo et al. 2016, Machine Intelligence Garage's Ethics Committee 2018, Leslie 2019, Food and Drug Administration 2020, Gebru et al. 2020, High-Level Expert Group on Artificial Intelligence 2020, Mongan et al. 2020, Pineau 2020, Riley et al. 2020, UK Department of Health & Social Care 2021, Weikert et al. 2021
	Representativeness	Diakopoulos et al., Bossuyt et al. 2003, Food and Drug Administration 2012, Collins et al. 2015, Luo et al. 2016, Global Future Council on Human Rights 2018, High-Level Expert Group on Artificial Intelligence 2018, Machine Intelligence Garage's Ethics Committee 2018, Park and Han 2018, Matheny et al. 2019, Benjamins et al. 2019, Cearns et al. 2019, Leslie 2019, Magrabi et al. 2019, Monetary Authority of Singapore 2019, Turner Lee et al. 2019, Wang and Preininger 2019, Wiens et al. 2019, Arrieta et al. 2020, Food and Drug Administration 2020, Gebru et al. 2020, High-Level Expert Group on Artificial Intelligence 2020, Information Commissioner's Office 2020a, Liu, Rivera et al. 2020, Mongan et al. 2020, Norgeot et al. 2020, Pineau 2020, Riley et al. 2020, Rivera, Liu et al. 2020, Shneiderman 2020, Stevens et al. 2020, Vollmer 2020, Ryan and Stahl 2021, UK Department of Health & Social Care 2021, Weikert et al. 2021
	Data quality	Diakopoulos et al., Bossuyt et al. 2003, Bhaskar et al. 2006, Nykänen et al. 2011, Food and Drug Administration 2012, Steyerberg and Vergouwe 2014, Alonso 2015, Collins et al. 2015, Luo et al. 2016, Global Future Council on Human Rights 2018, High-Level Expert Group on Artificial Intelligence 2018, Kappen et al. 2018, Machine Intelligence Garage's Ethics Committee 2018, Park and Han 2018, Matheny et al. 2019, Cearns et al. 2019, Leslie 2019, Magrabi et al. 2019, Turner Lee et al. 2019, Arrieta et al. 2020, Food and Drug Administration 2020, Gebru et al. 2020, High-Level Expert Group on Artificial Intelligence 2020, Liaw et al. 2020, Liu, Rivera et al. 2020, Mongan et al. 2020, Norgeot et al. 2020, Pineau 2020, Rivera, Liu et al. 2020, Serban et al. 2020, Shneiderman 2020, Stevens et al. 2020, Vollmer 2020, Larson et al. 2021, Ryan and Stahl 2021, UK Department of Health & Social Care 2021, Weikert et al. 2021
	Data preprocessing	Diakopoulos et al., Bhaskar et al. 2006, Steyerberg 2009, Food and Drug Administration 2012, Steyerberg and Vergouwe 2014, Collins et al. 2015, Luo et al. 2016, High-Level Expert Group on Artificial Intelligence 2018, Kappen et al. 2018, Matheny et al. 2019, Cearns et al. 2019, Leslie 2019, Food and Drug Administration 2020, Liu, Rivera et al. 2020, Mongan et al. 2020, Norgeot et al. 2020, Pineau 2020, Poldrack et al. 2020, Rivera, Liu et al. 2020, Stevens et al. 2020
	Data coding standards	High-Level Expert Group on Artificial Intelligence 2020, UK Department of Health & Social Care 2021

Phase 2	Model selection and interpretability	Diakopoulos et al., Bhaskar et al. 2006, Steyerberg and Vergouwe 2014, Sculley et al. 2015, Luo et al. 2016, Datatilsynet 2018, Global Future Council on Human Rights 2018, High-Level Expert Group on Artificial Intelligence 2018, Matheny et al. 2019, Cearns et al. 2019, Kelly et al. 2019, Leslie 2019, Magrabi et al. 2019, Miller 2019, Molnar 2019, Arrieta et al. 2020, High-Level Expert Group on Artificial Intelligence 2020, Huang et al. 2020, Information Commissioner's Office 2020a, Information Commissioner's Office 2020b, Liaw et al. 2020, Mongan et al. 2020, Norgeot et al. 2020, Sartor and Lagioia 2020, Stevens et al. 2020, Vollmer 2020, Larson et al. 2021, Ryan and Stahl 2021, UK Department of Health & Social Care 2021, Weikert et al. 2021
	Training the AIPM	Diakopoulos et al., Aliferis et al. 2007, Food and Drug Administration 2012, Collins et al. 2015, Matheny et al. 2019, Cearns et al. 2019, Eggensperger et al. 2019, Mongan et al. 2020, Pineau 2020, Serban et al. 2020, Stevens et al. 2020, Weikert et al. 2021
	Measures to reduce risk of overfitting	Bhaskar et al. 2006, Aliferis et al. 2007, Altman et al. 2009, Steyerberg 2009, Food and Drug Administration 2012, Steyerberg and Vergouwe 2014, Collins et al. 2015, Luo et al. 2016, Park and Han 2018, Cearns et al. 2019, Eggensperger et al. 2019, Kaur et al. 2019, Kelly et al. 2019, Van Calster et al. 2019, Norgeot et al. 2020, Stevens et al. 2020, Weikert et al. 2021
	Measures to identify and prevent algorithmic bias	Steyerberg and Vergouwe 2014, Global Future Council on Human Rights 2018, High-Level Expert Group on Artificial Intelligence 2018, Park and Han 2018, Benjamins et al. 2019, Cearns et al. 2019, Kelly et al. 2019, Leslie 2019, Turner Lee et al. 2019, Wang and Preininger 2019, Wiens et al. 2019, Arrieta et al. 2020, Food and Drug Administration 2020, High-Level Expert Group on Artificial Intelligence 2020, Information Commissioner's Office 2020a, Shneiderman 2020, Vollmer 2020, Google AI 2021, Ryan and Stahl 2021, UK Department of Health & Social Care 2021, Weikert et al. 2021
	Internal validation	Diakopoulos et al., Bossuyt et al. 2003, Altman et al. 2009, Moons et al. 2009, Steyerberg 2009, Food and Drug Administration 2012, Steyerberg and Vergouwe 2014, Luo et al. 2016, Food and Drug Administration 2017, Global Future Council on Human Rights 2018, Kappen et al. 2018, Park and Han 2018, Matheny et al. 2019, Cearns et al. 2019, Eggensperger et al. 2019, Kelly et al. 2019, Leslie 2019, Magrabi et al. 2019, Van Calster et al. 2019, Wiens et al. 2019, Food and Drug Administration 2020, Mongan et al. 2020, Norgeot et al. 2020, Pineau 2020, Poldrack et al. 2020, Stevens et al. 2020, Vollmer 2020, Google AI 2021, Larson et al. 2021, Weikert et al. 2021
	Transparency of the modelling process	Food and Drug Administration 2012, Collins et al. 2015, Global Future Council on Human Rights 2018, High-Level Expert Group on Artificial Intelligence 2018, Cearns et al. 2019, Turner Lee et al. 2019, Wiens et al. 2019, Liaw et al. 2020, Liu, Rivera et al. 2020, Mongan et al. 2020, Norgeot et al. 2020, Pineau 2020, Rivera, Liu et al. 2020, Serban et al. 2020, Stevens et al. 2020, Vollmer 2020, UK Department of Health & Social Care 2021, Weikert et al. 2021
Phase 3	Validation of the AIPM	Altman et al. 2009, Moons et al. 2009, Steyerberg 2009, Food and Drug Administration 2012, Moons et al. 2012, Steyerberg and Vergouwe 2014, Collins et al. 2015, Luo et al. 2016, Food and Drug Administration 2017, Kappen et al. 2018, Park and Han 2018, Matheny et al. 2019, Cearns et al. 2019, Kelly et al. 2019, Wiens et al. 2019, Mongan et al. 2020, Norgeot et al. 2020, Stevens et al. 2020, Vollmer 2020, Larson et al. 2021, Weikert et al. 2021
	Generalizability	Diakopoulos et al., Moons et al. 2009, Food and Drug Administration 2012, Steyerberg and Vergouwe 2014, Collins et al. 2015, Luo et al. 2016, Global Future Council on Human Rights 2018, Kappen et al. 2018, National Institute for Health and Care Excellence 2018, Park and Han 2018, Matheny et al. 2019, Benjamins et al. 2019, Berscheid and Roewer-Despres 2019, Cearns et al. 2019, Eggensperger et al. 2019, Kelly et al. 2019, Van Calster et al. 2019, High-Level Expert Group on Artificial Intelligence 2020, Information Commissioner's Office 2020a, Liu, Rivera et al. 2020, McCradden et al. 2020, Mongan et al. 2020, Norgeot et al. 2020, Serban et al. 2020, Stevens et al. 2020, Vollmer 2020, Google AI 2021, Larson et al. 2021,

		Weikert et al. 2021
Phase 4	Interoperability	Matheny et al. 2019, Ray et al. 2019, High-Level Expert Group on Artificial Intelligence 2020, UK Department of Health & Social Care 2021
	Human-AI interaction	Diakopoulos et al., eHealth Observatory 2012, Moons et al. 2012, Datatilsynet 2018, Global Future Council on Human Rights 2018, High-Level Expert Group on Artificial Intelligence 2018, Kappen et al. 2018, Machine Intelligence Garage's Ethics Committee 2018, Matheny et al. 2019, Amershi et al. 2019, Benjamins et al. 2019, Berscheid and Roewer-Despres 2019, Leslie 2019, Monetary Authority of Singapore 2019, Ray et al. 2019, Arrieta et al. 2020, High-Level Expert Group on Artificial Intelligence 2020, Information Commissioner's Office 2020a, Information Commissioner's Office 2020b, Sartor and Lagioia 2020, Sendak et al. 2020, Google AI 2021, Larson et al. 2021, Ryan and Stahl 2021, Smith et al. 2021, UK Department of Health & Social Care 2021, Weikert et al. 2021
	Facilitating software updating and monitoring	Diakopoulos et al., High-Level Expert Group on Artificial Intelligence 2018, Amershi et al. 2019, Leslie 2019, High-Level Expert Group on Artificial Intelligence 2020, Serban et al. 2020, Shneiderman 2020, Google AI 2021, Larson et al. 2021, Ryan and Stahl 2021, UK Department of Health & Social Care 2021
	Security	Diakopoulos et al., Datatilsynet 2017, Datatilsynet 2018, Machine Intelligence Garage's Ethics Committee 2018, Matheny et al. 2019, Benjamins et al. 2019, Cearns et al. 2019, Leslie 2019, Wang and Preininger 2019, Arrieta et al. 2020, High-Level Expert Group on Artificial Intelligence 2020, Information Commissioner's Office 2020a, Liaw et al. 2020, Rodríguez et al. 2020, Google AI 2021, Larson et al. 2021, Ryan and Stahl 2021, UK Department of Health & Social Care 2021
	Software testing	Datatilsynet 2017, High-Level Expert Group on Artificial Intelligence 2018, National Institute for Health and Care Excellence 2018, Food and Drug Administration 2020, High-Level Expert Group on Artificial Intelligence 2020, Information Commissioner's Office 2020a, Serban et al. 2020, Shneiderman 2020, Google AI 2021, Larson et al. 2021
Phase 5	Feasibility study	Moons et al. 2009, Nykänen et al. 2011, Kappen et al. 2018, Machine Intelligence Garage's Ethics Committee 2018, National Institute for Health and Care Excellence 2018, Cearns et al. 2019, Kelly et al. 2019, Ray et al. 2019, Wiens et al. 2019, Food and Drug Administration 2020, Liu, Rivera et al. 2020, Rivera, Liu et al. 2020, Larson et al. 2021, Smith et al. 2021
	Impact study	Moons et al. 2009, Steyerberg 2009, Nykänen et al. 2011, Moons et al. 2012, Global Future Council on Human Rights 2018, Kappen et al. 2018, Machine Intelligence Garage's Ethics Committee 2018, National Institute for Health and Care Excellence 2018, Park and Han 2018, Matheny et al. 2019, Cearns et al. 2019, Kelly et al. 2019, Magrabi et al. 2019, Wiens et al. 2019, Food and Drug Administration 2020, Liu, Rivera et al. 2020, McCradden et al. 2020, Rivera, Liu et al. 2020, Vollmer 2020, UK Department of Health & Social Care 2021, Weikert et al. 2021
	Risk management	Diakopoulos et al., Nykänen et al. 2011, Global Future Council on Human Rights 2018, High-Level Expert Group on Artificial Intelligence 2018, Machine Intelligence Garage's Ethics Committee 2018, Magrabi et al. 2019, Arrieta et al. 2020, High-Level Expert Group on Artificial Intelligence 2020, Liu, Rivera et al. 2020, Rivera, Liu et al. 2020, Shneiderman 2020, Vollmer 2020, UK Department of Health & Social Care 2021
Phase 6	Clinical implementation	Global Future Council on Human Rights 2018, High-Level Expert Group on Artificial Intelligence 2018, Kappen et al. 2018, National Institute for Health and Care Excellence 2018, Benjamins et al. 2019, Berscheid and Roewer-Despres 2019, Kelly et al. 2019, Monetary Authority of Singapore 2019, Ray et al. 2019, Wang and Preininger 2019, Wiens et al. 2019, High-Level Expert Group on Artificial Intelligence 2020, Information Commissioner's Office 2020a, McCradden et al. 2020, Serban et al. 2020, Vollmer 2020, Google AI 2021, Larson et al. 2021, Smith et al. 2021, UK Department of Health & Social Care 2021, Weikert et al. 2021

	Maintenance and updating	Sculley et al. 2015, Wiens et al. 2019, Information Commissioner's Office 2020a, Larson et al. 2021
	Education	Food and Drug Administration 2012, Datatilsynet 2017, Global Future Council on Human Rights 2018, High-Level Expert Group on Artificial Intelligence 2018, Kappen et al. 2018, Machine Intelligence Garage's Ethics Committee 2018, Benjamins et al. 2019, Berscheid and Roewer-Despres 2019, Leslie 2019, Magrabi et al. 2019, High-Level Expert Group on Artificial Intelligence 2020, Information Commissioner's Office 2020a, McCradden et al. 2020, Mongan et al. 2020, Shneiderman 2020, UK Department of Health & Social Care 2021
	Monitoring and auditing	Diakopoulos et al., Moons et al. 2009, Sculley et al. 2015, Datatilsynet 2017, Food and Drug Administration 2017, Global Future Council on Human Rights 2018, High-Level Expert Group on Artificial Intelligence 2018, Machine Intelligence Garage's Ethics Committee 2018, National Institute for Health and Care Excellence 2018, Matheny et al. 2019, Cearns et al. 2019, Leslie 2019, Magrabi et al. 2019, Monetary Authority of Singapore 2019, Turner Lee et al. 2019, Food and Drug Administration 2020, High-Level Expert Group on Artificial Intelligence 2020, Information Commissioner's Office 2020a, Liu, Rivera et al. 2020, McCradden et al. 2020, Serban et al. 2020, Shneiderman 2020, Vollmer 2020, Google AI 2021, Larson et al. 2021, Ryan and Stahl 2021, UK Department of Health & Social Care 2021

Supplementary Table 7. Source per included reference

Reference	Affiliation(s)	Geographical region	Type of source
Aliferis, C.F., A. Statnikov, and I. Tsamardinos, *Challenges in the analysis of mass-throughput data: a technical commentary from the statistical machine learning perspective.* Cancer Inform, 2007. **2**: p. 133-62.	Academia	USA	Literature search
Alonso, O., *Challenges with Label Quality for Supervised Learning.* Acm Journal of Data and Information Quality, 2015. **6**(1).	Industry, Academia	USA	Literature search
Amershi, S., D. Weld, M. Vorvoreanu, et al., *Guidelines for Human-AI Interaction*, in *Proceedings of the 2019 CHI Conference on Human Factors in Computing Systems*. 2019, Association for Computing Machinery: Glasgow, Scotland Uk. p. Paper 3.	Industry, Academia	USA	Literature search
Arrieta, A.B., N. Diaz-Rodriguez, J. Del Ser, et al., *Explainable Artificial Intelligence (XAI): Concepts, taxonomies, opportunities and challenges toward responsible AI.* Information Fusion, 2020. **58**: p. 82-115.	Industry, Academia	Spain, France	Literature search
Berscheid, J. and F. Roewer-Despres, *Beyond transparency: a proposed framework for accountability in decision-making AI systems.* AI Matters, 2019. **5**(2): p. 13–22.	Academia	Canada	Literature search
Bhaskar, H., D.C. Hoyle, and S. Singh, *Machine learning in bioinformatics: a brief survey and recommendations for practitioners.* Comput Biol Med, 2006. **36**(10): p. 1104-25.	Academia	United Kingdom	Literature search
Cearns, M., T. Hahn, and B.T. Baune, *Recommendations and future directions for supervised machine learning in psychiatry.* Transl Psychiatry, 2019. **9**(1): p. 271.	Academia	Australia, Germany	Literature search
Collins, G.S., J.B. Reitsma, D.G. Altman, and K.G.M. Moons, *Transparent Reporting of a Multivariable Prediction Model for Individual Prognosis or Diagnosis (TRIPOD): The TRIPOD Statement.* Eur Urol, 2015. **67**(6): p. 1142-1151.	Academia	The Netherlands, UK, Australia, USA	Literature search
Eggensperger, K., M. Lindauer, and F. Hutter, *Pitfalls and best practices in algorithm configuration.* J. Artif. Int. Res., 2019. **64**(1): p. 861–893.	Academia	Germany	Literature search
Huang, S.C., A. Pareek, S. Seyyedi, I. Banerjee, and M.P. Lungren, *Fusion of medical imaging and electronic health records using deep learning: a systematic review and implementation guidelines.* NPJ Digit Med, 2020. **3**: p. 136.	Academia	USA	Literature search
Kaur, H., H.S. Pannu, and A.K. Malhi, *A Systematic Review on Imbalanced Data Challenges in Machine Learning: Applications and Solutions.* Acm Computing Surveys, 2019. **52**(4).	Academia	India	Literature search

Kelly, C.J., A. Karthikesalingam, M. Suleyman, G. Corrado, and D. King, *Key challenges for delivering clinical impact with artificial intelligence.* BMC Med, 2019. **17**(1): p. 195.	Industry	United Kingdom, USA	Literature search
Larson, D.B., H. Harvey, D.L. Rubin, N. Irani, J.R. Tse, and C.P. Langlotz, *Regulatory Frameworks for Development and Evaluation of Artificial Intelligence Based Diagnostic Imaging Algorithms: Summary and Recommendations.* Journal of the American College of Radiology, 2021. **18**(3): p. 413-424.	Academia	USA, United Kingdom	Literature search
Liaw, S.T., H. Liyanage, C. Kuziemsky, et al., *Ethical Use of Electronic Health Record Data and Artificial Intelligence: Recommendations of the Primary Care Informatics Working Group of the International Medical Informatics Association.* Yearb Med Inform, 2020. **29**(1): p. 51-57.	Academia	Australia, United Kingdom, Canada, USA	Literature search
Liu, X., S.C. Rivera, D. Moher, M.J. Calvert, and A.K. Denniston, *Reporting guidelines for clinical trial reports for interventions involving artificial intelligence: the CONSORT-AI Extension.* Bmj, 2020. **370**: p. m3164.	Academia	United Kingdom, Canada, Swiss, USA, Australia	Literature search
Luo, W., D. Phung, T. Tran, et al., *Guidelines for Developing and Reporting Machine Learning Predictive Models in Biomedical Research: A Multidisciplinary View.* Journal of Medical Internet Research, 2016. **18**(12).	Industry, Academia	Australia, USA, Japan	Literature search
Magrabi, F., E. Ammenwerth, J.B. McNair, et al., *Artificial Intelligence in Clinical Decision Support: Challenges for Evaluating AI and Practical Implications.* Yearb Med Inform, 2019. **28**(1): p. 128-134.	Academia	Australia, Austria, Denmark, The Netherlands, Finland, United Kingdom, Japan	Literature search
McCradden, M.D., S. Joshi, J.A. Anderson, M. Mazwi, A. Goldenberg, and R. Zlotnik Shaul, *Patient safety and quality improvement: Ethical principles for a regulatory approach to bias in healthcare machine learning.* J Am Med Inform Assoc, 2020. **27**(12): p. 2024-2027.	Academia	Canada	Literature search
Rivera, S.C., X.X. Liu, A.W. Chan, et al., *Guidelines for clinical trial protocols for interventions involving artificial intelligence: the SPIRIT-AI extension.* Nature Medicine, 2020. **26**(9): p. 1351-1363.	Academia	United Kingdom, Canada	Literature search
Rodríguez, N., G. Stipcich, D. Jiménez, et al., *Federated Learning and Differential Privacy: Software tools analysis, the Sherpa.ai FL framework and methodological guidelines for preserving data privacy.* Information Fusion, 2020. **64**.	Academia	Spain	Literature search
Ryan, M. and B.C. Stahl, *Artificial intelligence ethics guidelines for developers and users: clarifying their content and normative implications.* Journal of Information, Communication and Ethics in Society, 2021. **19**(1): p. 61-86.	Academia	Sweden, United Kingdom	Literature search

Serban, A., K.v.d. Blom, H. Hoos, and J. Visser, *Adoption and Effects of Software Engineering Best Practices in Machine Learning*, in *Proceedings of the 14th ACM / IEEE International Symposium on Empirical Software Engineering and Measurement (ESEM)*. 2020, Association for Computing Machinery: Bari, Italy. p. Article 3.	Academia	The Netherlands	Literature search
Shneiderman, B., *Bridging the Gap Between Ethics and Practice: Guidelines for Reliable, Safe, and Trustworthy Human-centered AI Systems.* ACM Trans. Interact. Intell. Syst., 2020. **10**(4): p. Article 26.	Academia	USA	Literature search
Smith, M., A. Sattler, G. Hong, and S. Lin, *From Code to Bedside: Implementing Artificial Intelligence Using Quality Improvement Methods.* Journal of general internal medicine, 2021. **36**(4): p. 1061-1066.	Academia	USA	Literature search
Stevens, L.M., B.J. Mortazavi, R.C. Deo, L. Curtis, and D.P. Kao, *Recommendations for Reporting Machine Learning Analyses in Clinical Research.* Circ Cardiovasc Qual Outcomes, 2020. **13**(10): p. e006556.	Academia	USA	Literature search
Wang, F. and A. Preininger, *AI in Health: State of the Art, Challenges, and Future Directions.* Yearb Med Inform, 2019. **28**(1): p. 16-26.	Industry, Academia	USA	Literature search
Weikert, T., M. Francone, S. Abbara, et al., *Machine learning in cardiovascular radiology: ESCR position statement on design requirements, quality assessment, current applications, opportunities, and challenges.* European Radiology, 2021. **31**(6): p. 3909-3922.	Academia	Swiss, Italy, USA, South Korea, Germany, Austria, France, United Kingdom, Belgium, The Netherlands	Literature search
Benjamins, R., A. Barbado, and D. Sierra. *Responsible AI by Design in Practice*. in *AAAI Fall Symposium*. 2019.	Industry	Spain	Snowballing
Datatilsynet, *Software development with Data Protection by Design and by Default*. 2017, The Norwegian Data Protection Authority.	Governing	Norway	Snowballing
Datatilsynet, *Artificial intelligence and privacy*. 2018, The Norwegian Data Protection Authority.	Governing	Norway	Snowballing
Diakopoulos, N., S. Friedler, M. Arenas, et al. *Principles for Accountable Algorithms and a Social Impact Statement for Algorithms*. Available from: https://www.fatml.org/resources/principles-for-accountable-algorithms.	Industry, Academia	USA, United Kingdom, Chile	Snowballing
eHealth Observatory, *Canada health infoway benefits evaluation indicators*. 2012.	Governing	Canada	Snowballing

Gebru, T., J. Morgenstern, B. Vecchione, et al., *Datasheets for Datasets.* 2020.	Industry, Academia	USA	Snowballing
Global Future Council on Human Rights 2016-2018, *How to prevent discriminatory outcomes in machine learning*. 2018, World Economic Forum.	Governing	Global	Snowballing
Google AI. *Responsible AI practices*. 2021; Available from: https://ai.google/responsibilities/responsible-ai-practices/.	Industry	USA	Snowballing
High-Level Expert Group on Artificial Intelligence, *Draft ethics guidelines for trustworthy AI*. 2018, European Commission.	Governing	Europe	Snowballing
High-Level Expert Group on Artificial Intelligence, *The assessment list for trustworthy artificial intelligence (ALTAI) for self-assessment*. 2020, European Commission.	Governing	Europe	Snowballing
Leslie, D., *Understanding artificial intelligence ethics and safety: A guide for the responsible design and implementation of AI systems in the public sector.* 2019, The Alan Turing Institute.	Governing, Academia	United Kingdom	Snowballing
Machine Intelligence Garage's Ethics Committee, *Ethics Framework*. 2018, Digital Catapult.	Industry	United Kingdom	Snowballing
Miller, T., *Explanation in artificial intelligence: Insights from the social sciences.* Artificial Intelligence, 2019. **267**: p. 1-38.	Academia	Australia	Snowballing
Monetary Authority of Singapore, *Principles to Promote Fairness, Ethics, Accountability and Transparency (FEAT) in the Use of Artificial Intelligence and Data Analytics in Singapore's Financial Sector*. 2019.	Governing	Singapore	Snowballing
Norgeot, B., G. Quer, B.K. Beaulieu-Jones, et al., *Minimum information about clinical artificial intelligence modeling: the MI-CLAIM checklist.* Nature Medicine, 2020. **26**(9): p. 1320-1324.	Industry, Academia	USA	Snowballing
Nykänen, P., J. Brender, J. Talmon, et al., *Guideline for good evaluation practice in health informatics (GEP-HI).* International Journal of Medical Informatics, 2011. **80**(12): p. 815-827.	Academia	Finland, Denmark, The Netherlands, United Kingdom, France, Austria	Snowballing
Pineau, J., P. Vincent-Lamarre, K. Sinha, et al., *The Machine Learning Reproducibility Checklist*, in *Neural Information Processing Systems*. 2020.	Industry, Academia	Canada, USA	Snowballing
Poldrack, R.A., G. Huckins, and G. Varoquaux, *Establishment of Best Practices for Evidence for Prediction: A Review.* JAMA Psychiatry, 2020. **77**(5): p. 534-540.	Academia, Governing	USA, France	Snowballing
Ray, J.M., R.M. Ratwani, C.A. Sinsky, et al., *Six habits of highly*	Industry, Academia	USA	Snowballing

successful health information technology: powerful strategies for design and implementation. Journal of the American Medical Informatics Association, 2019. **26**(10): p. 1109-1114.			
Riley, R.D., J. Ensor, K.I.E. Snell, et al., *Calculating the sample size required for developing a clinical prediction model.* BMJ, 2020. **368**: p. m441.	Academia	USA, United Kingdom, The Netherlands	Snowballing
Sculley, D., G. Holt, D. Golovin, et al., *Hidden technical debt in Machine learning systems*, in *Proceedings of the 28th International Conference on Neural Information Processing Systems - Volume 2*. 2015, MIT Press: Montreal, Canada. p. 2503–2511.	Industry	USA	Snowballing
Turner Lee, N., P. Resnick, and G. Barton. *Algorithmic bias detection and mitigation: Best practices and policies to reduce consumer harms*. 2019; Available from: https://www.brookings.edu/research/algorithmic-bias-detection-and-mitigation-best-practices-and-policies-to-reduce-consumer-harms/	Governing, Academia	USA	Snowballing
Van Calster, B., D.J. McLernon, M. van Smeden, et al., *Calibration: the Achilles heel of predictive analytics.* BMC Medicine, 2019. **17**(1): p. 230.	Academia	Belgium, United Kingdom, The Netherlands	Snowballing
Altman, D.G., Y. Vergouwe, P. Royston, and K.G.M. Moons, *Prognosis and prognostic research: validating a prognostic model.* BMJ, 2009. **338**: p. b605.	Academia	United Kingdom, The Netherlands	Expert consultation
Artificial intelligence in health care: The hope, the hype, the promise, the peril, ed. M. Matheny, et al. 2019, Washington, DC: National Academy of Medicine.	Industry, Academia	USA	Expert consultation
Bossuyt, P.M., J.B. Reitsma, D.E. Bruns, et al., *Towards Complete and Accurate Reporting of Studies of Diagnostic Accuracy: The STARD Initiative.* Clinical Chemistry, 2003. **49**(1): p. 1-6.	Academia	The Netherlands, USA, Australia, Canada	Expert consultation
Food and Drug Administration, *Computer-Assisted Detection Devices Applied to Radiology Images and Radiology Device Data - Premarket Notification [510(k)] Submissions*. 2012.	Governing	USA	Expert consultation
Food and Drug Administration, *Software as a Medical Device (SAMD): Clinical Evaluation*. 2017.	Governing	USA	Expert consultation
Food and Drug Administration, *Clinical Performance Assessment: Considerations for Computer-Assisted Detection Devices Applied to Radiology Images and Radiology Device Data in Premarket Notification (510(k)) Submissions*. 2020.	Governing	USA	Expert consultation
Information Commissioner's Office, *Guidance on the AI auditing framework: Draft guidance for consultation*. 2020.	Governing	United Kingdom	Expert consultation
Information Commissioner's Office. *ICO and the Turing*	Governing,	United	Expert

consultation on explaining AI decisions guidance. 2020; Available from: https://ico.org.uk/about-the-ico/ico-and-stakeholder-consultations/ico-and-the-turing-consultation-on-explaining-ai-decisions-guidance/.	Academia	Kingdom	consultation
Kappen, T.H., W.A. van Klei, L. van Wolfswinkel, C.J. Kalkman, Y. Vergouwe, and K.G.M. Moons, *Evaluating the impact of prediction models: lessons learned, challenges, and recommendations.* Diagn Progn Res, 2018. **2**: p. 11.	Academia	The Netherlands	Expert consultation
Molnar, C., *Interpretable Machine Learning: A Guide for Making Black Box Models Explainable*. 2019.	Academia	Germany	Expert consultation
Mongan, J., L. Moy, and C.E. Kahn, *Checklist for Artificial Intelligence in Medical Imaging (CLAIM): A Guide for Authors and Reviewers.* Radiology: Artificial Intelligence, 2020. **2**(2): p. e200029.	Academia	USA	Expert consultation
Moons, K.G., D.G. Altman, Y. Vergouwe, and P. Royston, *Prognosis and prognostic research: application and impact of prognostic models in clinical practice.* Bmj, 2009. **338**: p. b606.	Academia	The Netherlands, United Kingdom	Expert consultation
Moons, K.G., A.P. Kengne, D.E. Grobbee, et al., *Risk prediction models: II. External validation, model updating, and impact assessment.* Heart, 2012. **98**(9): p. 691-8.	Academia	The Netherlands	Expert consultation
National Institute for Health and Care Excellence, *Evidence standards framework for digital health technologies*. 2018.	Governing	United Kingdom	Expert consultation
Park, S.H. and K. Han, *Methodologic Guide for Evaluating Clinical Performance and Effect of Artificial Intelligence Technology for Medical Diagnosis and Prediction.* Radiology, 2018. **286**(3): p. 800-809.	Academia	South Korea	Expert consultation
Sartor, G. and F. Lagioia, *The impact of the General Data Protection Regulation (GDPR) on artificial intelligence*. 2020, Panel for the Future of Science and Technology.	Governing, Academia	Europe	Expert consultation
Sendak, M.P., M. Gao, N. Brajer, and S. Balu, *Presenting machine learning model information to clinical end users with model facts labels.* npj Digital Medicine, 2020. **3**(1): p. 41.	Academia	USA	Expert consultation
Steyerberg, E.W., *Clinical Prediction Models*, ed. M. Gail, M.S. Jonathan, and B. Singer. 2009, Cham, Switzerland: Springer Nature.	Academia	The Netherlands	Expert consultation
Steyerberg, E.W. and Y. Vergouwe, *Towards better clinical prediction models: seven steps for development and an ABCD for validation.* Eur Heart J, 2014. **35**(29): p. 1925-31.	Academia	The Netherlands	Expert consultation
UK Department of Health & Social Care. *A guide to good practice for digital and data-driven health technologies*. 2021; Available from:	Governing	United Kingdom	Expert consultation

https://www.gov.uk/government/publications/code-of-conduct-for-data-driven-health-and-care-technology/initial-code-of-conduct-for-data-driven-health-and-care-technology.			
Vollmer, S., B.A. Mateen, G. Bohner, et al., *Machine learning and artificial intelligence research for patient benefit: 20 critical questions on transparency, replicability, ethics, and effectiveness.* Bmj-British Medical Journal, 2020. **368**.	Governing, Academia	United Kingdom, USA, The Netherlands	Expert consultation
Wiens, J., S. Saria, M. Sendak, et al., *Do no harm: a roadmap for responsible machine learning for health care.* Nat Med, 2019. **25**(9): p. 1337-1340.	Industry, Academia	USA, Canada	Expert consultation
